# Uveitis characteristics and multiple sclerosis phenotype of patients with multiple sclerosis-associated uveitis: A systematic review and meta-analysis

**DOI:** 10.1371/journal.pone.0307455

**Published:** 2024-10-25

**Authors:** Paola Saboya-Galindo, Germán Mejía-Salgado, Carlos Cifuentes-González, Camilo Andrés Rodríguez-Rodríguez, Laura Boada-Robayo, Rafael Méndez-Marulanda, Joan Sebastián Varela, Laura Riveros-Sierra, Mariana Gaviria-Carrillo, Alejandra de-la-Torre

**Affiliations:** 1 Ophthalmology Interest Group-Universidad del Rosario (OIG UR), Neuroscience (NEUROS) Research Group, Neurovitae Research Center, Institute of Translational Medicine (IMT), Escuela de Medicina y Ciencias de la Salud, Universidad del Rosario, Bogotá, Colombia; 2 Neuroscience (NEUROS) Research Group, Neurovitae Research Center, Institute of Translational Medicine (IMT), Escuela de Medicina y Ciencias de la Salud, Universidad del Rosario, Bogotá, Colombia; 3 Ophthalmology Department, Facultad de Medicina, Hospital Universitario San Ignacio, Pontificia Universidad Javeriana, Bogotá, Colombia; Irrua Specialist Teaching Hospital, NIGERIA

## Abstract

**Purpose:**

To summarize and meta-analyze uveitis characteristics and multiple sclerosis (MS) phenotype of patients with multiple sclerosis-associated uveitis (MSAU) within a systematic review and meta-analysis.

**Methods:**

A comprehensive literature search was performed on January 25, 2023, utilizing PubMed, Embase, and Virtual Health Library (VHL) databases. We included studies involving patients with MSAU, such as case series with over 10 patients, cross-sectional, case-control, and cohort studies. Quality and risk of bias were assessed using CLARITY tools and validated metrics like the Hoy et al. and Hassan Murad et al. tools. The pooled analysis focused on 1) uveitis characteristics, 2) ocular complications, 3) MS phenotype, and 3) administered treatments for uveitis and MS. Gender-based subgroup analysis was conducted across continents; heterogeneity was measured using the I2 statistic. Statistical analysis was performed using R software version 4.3.1. The study was registered in PROSPERO with CRD42023453495 number.

**Results:**

Thirty-six studies were analyzed (24 with a low risk of bias, 8 with some concerns, and 4 with a high risk of bias), including 1,257 patients and 2,034 eyes with MSAU. The pooled analysis showed a mean age of 38.2 ± 12.1 years with a notable female predominance (67%, 95% CI [59%-73%]). MS before uveitis was seen in 59% of the cases (95% CI [48%-69%]), while uveitis was present before MS in 38% (95% CI [30%-48%]). The mean age for the first uveitis episode was 35.7 ± 8.3 years, predominantly affecting both eyes (77%, 95% CI [69%-83%], from 23 studies involving 452 patients). Intermediate uveitis was the most frequent anatomical location (68%, 95% CI [49%-82%], from 22 studies involving 530 patients), often following a recurrent course (63%, 95% CI [38%-83%]). Key complications included vision reduction (42%, 95% CI [19%-70%], from five articles involving 90 eyes), macular compromise (45%, 95% CI [20%-73%], from 4 studies involving 95 eyes), and cataracts (46%, 95% CI [32%-61%], from eight articles involving 230 eyes). Concerning MS phenotype, relapsing-remitting MS (RRMS) was the most common subtype (74%, 95% CI [64%-82%], from eight articles involving 134 patients), followed by secondary progressive MS (24%, 95% CI [18%-33%], from eight articles involving 125 patients). The most frequently occurring central nervous lesions were supratentorial (95%, 95% CI [70%-99%], from two articles involving 17 patients) and spinal cord (39%, 95% CI [16%-68%], from two articles involving 29 patients). The mean Expanded Disability Status Scale (EDSS) score and annual recurrence rates were 2.9 ± 0.6 and 1.07 ± 0.56, respectively. Treatment trends showed the prevalent use of Fingolimod (96%, 95% CI [17%-100%], from two articles involving 196 patients), Mycophenolate (48%, 95% CI [11%-87%], from four articles involving 51 patients), and Interferon-beta (43%, 95% CI [24%-65%], from 11 articles involving 325 patients).

**Conclusion:**

MSAU primarily affects young adult females, typically presenting as bilateral intermediate uveitis with vision-related complications. The most common MS phenotype is RRMS, often associated with supratentorial and spinal cord lesions on imaging. These findings give ophthalmologists and neurologists a comprehensive clinical picture of MSAU, facilitating prompt diagnosis.

## Introduction

Multiple sclerosis (MS) is a chronic inflammatory disorder of the central nervous system (CNS) that primarily affects white matter and is a leading cause of neurological disability in young adults, with a higher prevalence in females (3:1 female-to-male ratio) with a prevalence rate of 35 per 100,000 showing a decreasing gradient from north to south hemisphere [1, 2].

There are three types of MS. Relapsing-remitting MS (RRMS), accounting for 85% of all cases, consists of relapses that may leave sequelae, which remain stable between episodes. Secondary progressive MS (SPMS) follows RRMS and is characterized by a progressive phase following the remitting phase. Lastly, primary progressive MS (PPMS) is characterized by progression from the onset without a relapse phase, counting for 15% of the cases [3].

Optic neuritis is the most common ophthalmic manifestation of MS [4–6]; however, uveitis can also occur in approximately 1%-3% of MS patients (10 times higher than that in the general population). Furthermore, MS accounts for 1% of uveitis cases [7–9]. Initially, uveitis can present with mild vision impairment. Still, the visual prognosis can deteriorate due to common ocular complications such as cystoid macular edema, cataracts, glaucoma, vitreous hemorrhage, and occlusive vasculitis [10–12].

Bilateral intermediate uveitis (IU) is the most frequently described form in the literature. However, there is inconsistency across studies [10, 13]. The low prevalence of the disease means that most of the literature comes from observational studies with limited sample sizes and generally from a single center, which may not reflect the real clinical picture [7]. This systematic review aims to summarize and meta-analyze the characteristics of uveitis and MS phenotype of patients with multiple sclerosis-associated uveitis (MSAU).

## Materials and methods

### Type and design of the study

A systematic review and meta-analysis was conducted following the ’Preferred Reporting Items for Systematic Review and Meta-analysis’ PRISMA) statement **(S1 Checklist)**. This review was registered in PROSPERO under the reference CRD42023453495. Institutional review board approval was not required, as this study is based on data available in the public domain and did not use individual-level data.

### Search strategy

PubMed, Embase, and Virtual Health Library (VHL) were used to conduct a systematic literature search on January 25, 2023. The search algorithm included a combination of terms reflecting the diseases of interest (uveitis) and (multiple sclerosis) using the following search strategy: “multiple sclerosis”[MeSH Terms] OR “multiple sclerosis”[Title/Abstract]) AND (“Iridocyclitis”[MeSH Terms] OR “Iridocyclitis”[Title/Abstract] OR “Iritis”[MeSH Terms] OR “Iritis”[Title/Abstract] OR “Uveitis”[MeSH Terms] OR “Uveitis”[Title/Abstract] OR “retinal vasculitis”[MeSH Terms] OR “retinal vasculitis”[Title/Abstract]). The search strategies were modified to meet the criteria of each database **(S1 File)**.

### Study eligibility criteria

This systematic review considered primary studies involving at least 10 eyes or patients with MSAU. The accepted types of studies included case series defined as Hassan Murad [14], case-control studies, cohort studies, cross-sectional studies, and clinical trials. No restriction language or publication date was applied. Google Translate [15] was used to select the articles and extract the information for articles in languages other than English and Spanish. Excluded from consideration were non-full-text articles, studies conducted in species other than humans, case reports, economic analyses, systematic reviews, and secondary data sources.

Case series involving fewer than 10 eyes or patients for the specific variable were also excluded. This means that if the variable was measured at the level of the eye rather than the patient (e.g., location of uveitis, presence of synechiae) and it was 10 eyes or more, the study was included even if the number of patients was fewer than 10.

### Patient inclusion and exclusion criteria

In the current study, the inclusion criteria encompassed patients of all ethnicities, ages, and sexes, with a diagnosis of MS made according to the McDonald [16] or Poser criteria [17] and evidence of uveitis regardless of its location, onset, duration, and course. The patient was included when the article specified the development of uveitis at any point during the clinical course, whether before, simultaneously, or after the onset of MS. Patients with multiple sclerosis without uveitis were excluded.

The PROSPERO protocol was established to include only patients with diagnoses confirmed by McDonald criteria [16]. Nevertheless, as we do not establish a publication date restriction, we add Poser criteria [17] after PROSPERO registration to include patients with multiple sclerosis diagnosed before 2001.

### Study selection

All search results were uploaded to the Zotero® reference manager in RIS format. Subsequent steps included identifying and removing duplicate entries within Zotero®, complemented by verification in Microsoft Excel® using authors’ names, publication titles, and DOIs. After the duplicates were removed, the remaining studies were randomly assigned and evaluated independently by four pairs of reviewers, all trained in SUN, McDonald, and Poser criteria: LBR-CRR (100%), SGR-PSG (91.15%), SV-MG (86.76%), CHCG-GAMS (97.87%) with a concordance of 93.94%. This review process involved evaluating titles and abstracts, followed by a full-text assessment, with both stages adhering to predefined inclusion and exclusion criteria.

The outcomes of this review process led to articles being categorized as "included," "excluded," or "in doubt," documented in a Microsoft Excel® database. Disagreements that arose during the paired review were resolved through further discussion among the authors. In cases where consensus remained elusive, consultation with one uveitis specialist (AdlT) and one neurologist specialist (MG-C) was sought to reach a final decision.

### Data extraction

The included articles were coded and downloaded using the assigned code for information extraction. The identified papers were distributed among the eight reviewers (PS-G, GM-S, CAR-R, LBR, RM-M, JSV, LR-S, MG-C), who verified each article aligned with the predefined inclusion criteria.

Subsequently, the information extraction was carried out by two reviewers (PS-G, GM-S) systematically in a Microsoft Excel® spreadsheet, encompassing various key data points, including the article code, authorship details, article title, publication year, DOI, study design, the total number of patients and eyes studied, age at MS diagnosis, age at first episode of uveitis, number of patients who presented uveitis first to neurological signs, number of patients who presented MS before uveitis, clinical characteristics of uveitis (anatomical localization, onset, duration, clinical course, laterality, type of inflammation [granulomatous, non-granulomatous], slit-lamp findings [keratic precipitates, snowballs, snowbanks], ocular complications [cataracts, glaucoma, cystoid macular edema, epiretinal membrane, vitreous hemorrague] and treatment strategies employed.

### Risk of bias assessment

The risk of bias assessment was conducted independently by two reviewers (PS-G, GM-S), using validated tools depending on the methodological design of the article. The Clinical Advances Through Research and Information Translation (CLARITY) group contributed at McMaster University was used for cohort studies [18]. This scale evaluates 1) the selection of exposed and non-exposed cohorts, 2) the assessment of exposure, 3) the outcome of interest not present at the start of the study, 4) exposed and unexposed matching, 5) prognostic factors, 6) the assessment of outcome, 7) follow up, and 8) co-interventions. Cross-sectional studies used the Hoy et al. modified tool [19], consisting of 10 items addressing four domains plus a summary risk of bias assessment. The items are 1) study’s target population, 2) sampling frame representation, 3) sample selection, 4) likelihood of nonresponse, 5) data collection source, 6) case definition, 7) parameters measurement, 8) data collection consistency, 9) follow up period, and 10) appropriateness of numerator and denominator for the parameter of interest; the four domains are selection, nonresponse, measurement, and analysis bias. Case-control studies were assessed with CLARITY. This scale consists of 5 items: 1) assessment of exposure, 2) ascertainment of exposure, 3) selection of cases, 4) selection of controls, and 5) comparability and analysis of the data [20]. For the case series, we used Hassan Murad’s methodological quality scale [14]. It consists of 4 general items: 1) population selection, 2) ascertainment of exposure or outcome, 3) Causality, and 4) sufficient reporting details **(S2 File)**.

In cohort and case-control studies, we analyzed bias following the recommendations. However, to simplify the scoring (Definitely yes; Probably yes; Probably no; Definitely no), we assigned the following values: if the question was answered with ’Definitely yes,’ we assigned a ’low risk of bias.’ When it was ’Probably yes’ or ’Probably no,’ we used the term ’some concerns.’ If the classification was ’Definitely no,’ we assigned a ’high risk of bias.’ **(S2 File)**.

In cross-sectional studies, all yes accounts for one point; the external validity was rated ‘High’ for scores 0–1, ‘Some Concerns’ for score 2, and ‘Low’ for score 3. Similarly, internal validity was assessed as ‘High’ for scores 0–2, ‘Some Concerns’ for score 3, and ‘Low’ for scores 4. Ultimately, studies were considered to have a ‘High risk’ of bias if any domains (internal or external validity) or CLARITY questions received a ‘High risk of bias.’ **(S2 File)**.

In case-series studies, we analyzed bias following the recommendations; to simplify the scoring, we assigned the following values: if the question was answered ‘yes,’ we assigned a ‘Low risk of bias’; if the question was answered ‘no’ we assigned a ‘High risk of bias,’ and if the question was not applicable, we assigned ‘Some concerns. The figures were generated using Robvis, a visualization tool [21] **(S2 File)**.

### Data processing and analysis techniques

Statistical analyses were performed using R version 4.3.1 (R Foundation for Statistical Computing, Vienna, Austria). Meta-analyses were carried out on patients or eyes, depending on which had the most information, to perform proportion meta-analyses with a 95% confidence interval (CI) to construct forest plots for categorical data. The statistical heterogeneity of each study was assessed using the chi-square test and I2 statistics. The cutoff points for heterogeneity in the meta-analysis were 0%–30% low, 31%–50% moderate, and 51%–90% high [22]. Moreover, in order to improve the heterogeneity, subgroup analyses were conducted by geographic regions. A random effect model was performed in all metanalysis considering that most of the studies included will be observational studies in which a high heterogeneity is expected. Results were considered statistically significant if p<0.05. Finally, a pooled analysis was performed using continuous measures.

### Ethical statement

This study adheres to the ethical principles for human research established by the Helsinki Declaration, the Belmont Report, and Colombian Resolution 008430 of 1993.

## Results

### Study selection

**Fig 1:**
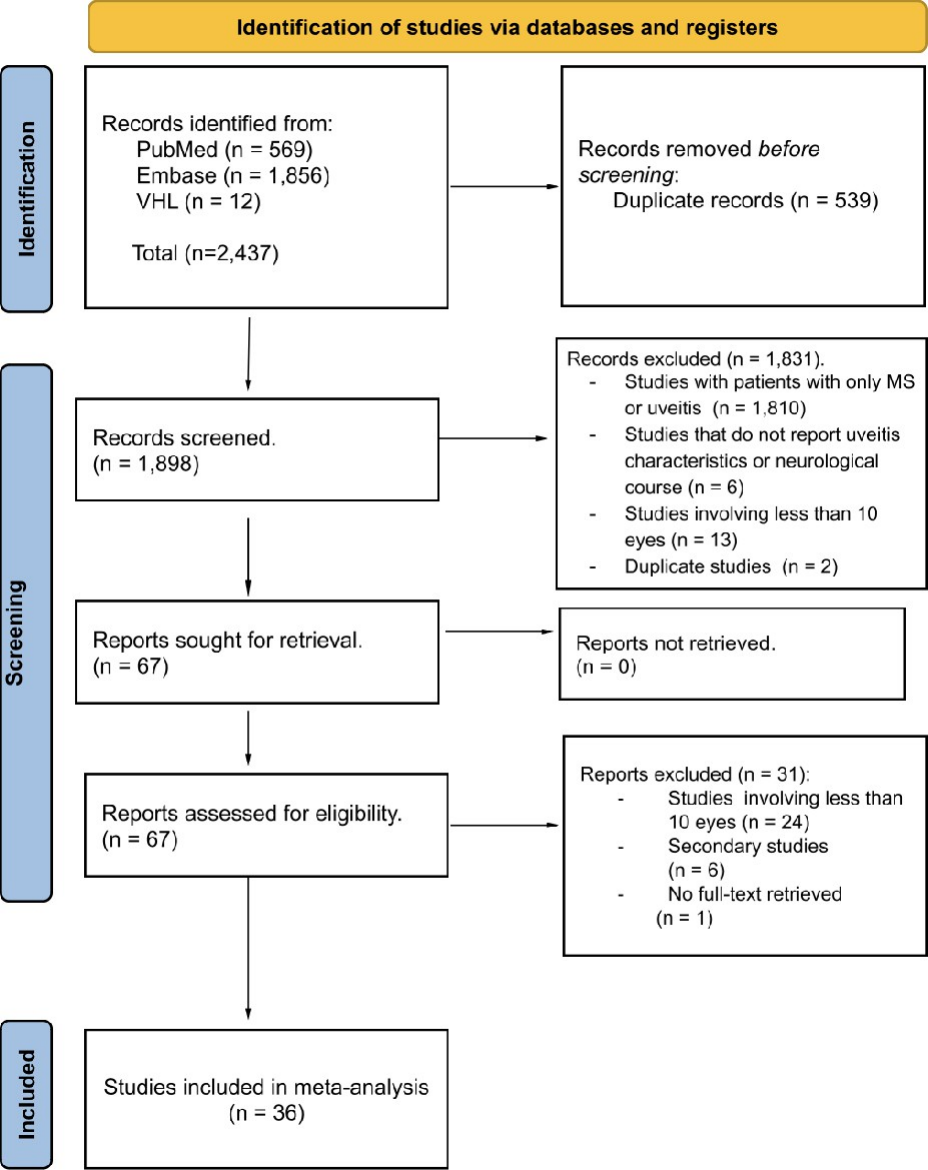
PRISMA flow diagram.

Initially, we identified 2,437 articles as potentially relevant studies. Among these, 539 duplicates were identified and subsequently eliminated. During the initial screening, 1,831 articles were excluded for various reasons: 1,810 focused solely on patients with either MS or uveitis, six did not report uveitis characteristics or neurological course, 13 involved studies with less than 10 eyes, and 2 were duplicate records. As a result, 67 articles were subjected to full-text review. However, 30 studies were excluded due to missing inclusion criteria, and one lacked full-text access. Consequently, 36 articles were included (**Fig 1).**

### Study characteristics

The included studies comprise 19 cross-sectional studies, nine case series, six cohort studies, and two case-control studies. Twelve cross-sectional studies were rated as having a ‘low risk of bias,’ while seven were categorized as having ‘some concerns.’ Regarding the case series, eight were assessed to have a ‘low risk of bias,’ with one identified as having a ‘high risk.’

Three cohort studies were determined to have a ‘high risk of bias,’ two were categorized as having a ‘low risk of bias,’ and one had ‘some concerns.’ The two case-control studies had a ‘low risk of bias.’

### Demographic characteristics of patients with multiple sclerosis-associated uveitis

This study included 1,257 patients (2,034 eyes) with MSAU. Based on data extracted from 15 articles, the average age of these patients was 38.2 years, with a standard deviation (SD) of 12.1. Five articles reporting the Expanded Disability Status Scale (EDSS) calculated the average EDSS to be 2.9 ± 0.6. Additionally, the average annual relapse rate, derived from three articles’ data, was 1.07 ± 0.56 per 12 months **(Table 1 and S3 File and S1 Raw data).**

**Table 1:** Baseline characteristics of multiple sclerosis and uveitis.

Author	Study design	Risk of bias	Cohort Size (Patients/Eyes)	Age of MSAU presentation (Mean ± SD)	Sex (M/F)	Outcomes of uveitis reported	Outcomes of MS reported	EDSS
EM Graham et al., 1989 [23]	Cross-sectional	Some concerns	9/17	28.3	5/4	Clinical characteristics	Clinical characteristics	NA
JI Lim et al., 1991[24]	Case series	High risk	6/12	43.8	0/6	Clinical characteristics	Clinical characteristics	NA
MA Acar et al., 1993 [25]	Case series	High risk	7/14	N/A	0/7	Clinical characteristics	Clinical characteristics	3.4
V Biousse et al., 1999 [26]	Cross-sectional	Low	28/50	47± N/A	8/20	Clinical characteristics	Clinical characteristics	NA
HMA Towler et al., 2000 [27]	Cross-sectional	Some concerns	16/21	37± 15	3/13	Clinical characteristics	Clinical characteristics	3 ± 2.3
S Schmidt et al., 2001 [28]	Cross-sectional	Some concerns	11/17	41.5 ± 9.4	7/4	Clinical characteristics and Treatment	Clinical characteristics	NA
JF Prieto et al., 2001 [29]	Cross-sectional	Some concerns	7/N/A	22.4± 11.5	N/A	Clinical characteristics	Clinical characteristics	NA
LJ Edwards et al., 2004 [30]	Case-control	Low	16/N/A	45 ± N/A	N/A	Clinical characteristics	Clinical characteristics and Treatment	NA
G Zein et al., 2004 [12]	Cross-sectional	Low	16/31	35.5	2/14	Clinical characteristics and Treatment	Clinical characteristics	NA
MD Becker et al., 2005 [31]	Case series	Some concerns	13/17	45.2 ± 7.4	5/8	Clinical characteristics/ Treatment	Clinical characteristics/ Treatment	NA
SM Maca et al 2006 [32]	Cross-sectional	Low risk	16/31	N/A	4/12	Clinical characteristics	Clinical characteristics	0–3
J Le Scanff et al., 2008 [33]	Cross-sectional	Some concerns	28/42	N/A	9/19	Clinical characteristics	Clinical characteristics	NA
LJ Edwards et al., 2008 [34]	Cohort	Low Risk	15/26	50	5/10	Clinical characteristics	Clinical characteristics	NA
E Jakob et al., 2009 [35]	Cross-sectional	Low risk	59/N/A	N/A	25/34	Clinical characteristics	Clinical characteristics	NA
Paović et al., 2009 [36]	Cross-sectional	Low risk	42/N/A	N/A	N/A	Clinical characteristics	Clinical characteristics	NA
A Langer-Gould et a., 2010 [37]	Case-control	Low risk	71/N/A	N/A	N/A	Clinical characteristics	Clinical characteristics	NA
MR Kanavi et al., 2010 [38]	Cross-sectional	Some concerns	4/N/A	32.5 6 11.2	N/A	Clinical characteristics	Clinical characteristics	NA
RA Marrie et al., 2011 [10]	Cohort	High Risk	N/A	40.7	N/A	Clinical characteristics	Clinical characteristics	NA
V Llorenç et al., 2012 [39]	Case series	Some concerns	7/17	N/A	2/5	Clinical characteristics/ Treatment	Clinical characteristics/ Treatment	NA
AM Karara et al., 2013 [40]	Cross sectional	Low Risk	7/8	32.64	5/2	Clinical characteristics/ Treatment	Clinical characteristics/ Treatment	NA
C Heinz et al., 2014 [41]	Cohort	Low Risk	23/N/A	23.8	N/A	Clinical characteristics	Clinical characteristics	NA
D Kaya et al., 2014 [11]	Cross-sectional	Low	9/16	42.0 ± 14.1	8/1	Clinical characteristics and Treatment	Clinical characteristics	NA
E Shugaiv et al., 2015 [42]	Cross-sectional	Low Risk	41/82	N/A	6/35	Clinical characteristics/ Treatment	Clinical characteristics/ Treatment	2
W Messenger et al., 2015 [43]	Cross-sectional	Low Risk	113/196	40.6	30/83	Clinical characteristics/ Treatment	Clinical characteristics	NA
D Velázquez et al., 2017 [44]	Case series	Some concerns	13/23	37.9 ± 9.5	6/7	Clinical characteristics/ Treatment	Clinical characteristics	3.8
A Hedayatfar et al., 2017 [45]	Case series	Some concerns	15/30	34.5± 8.3	1/14	Clinical characteristics	Clinical characteristics/ Treatment	NA
L Jouve et al., 2017 [46]	Cross-sectional	Some concerns	36/68	45± 8.8	11/25	Clinical characteristics/ Treatment	Clinical characteristics/ Treatment	NA
S Jovanović et al., 2017 [47]	Cross sectional	Low	25/30	40.5± 25.5	16/9	Clinical characteristics and Treatment	Clinical characteristics and Treatment	NA
LL Lim et al., 2019 [48]	Cross-sectional	Low Risk	189/N/A	41.9 ± 9.7	57/132	Clinical characteristics	Clinical characteristics/ Treatment	2.8 ± 1.5
TRP Taylor et al., 2020 [49]	Cross-sectional	Low Risk	8/N/A	N/A	1/7	Clinical characteristics	Clinical characteristics/ Treatment	NA
E Abd El Latif et al., 2020 [50]	Cohort	Some concerns	71/N/A	30.4 ± 3.1	N/A	Clinical characteristics	Clinical characteristics/ Treatment	NA
AF AlBloushi et al., 2021 [51]	Case series	Some concerns	20/38	29.5 ± 10.11	1/19	Clinical characteristics	Clinical characteristics	NA
AA Saifaldein et al., 2022 [52]	Case series	Some concerns	4/8	43.5	4/0	Clinical characteristics/ Treatment	Clinical characteristics/ Treatment	NA
NM Chirpaz et al., 2022 [53]	Cohort	High risk	10/17	35.8	3/7	Clinical characteristics/ Treatment	Clinical characteristics	NA
F Stascheit et al., 2022 [54]	Case series	Some concerns	6/12	N/A	1/5	Clinical characteristics	Clinical characteristics	NA
E Raskin et al., 2022 [55]	Cohort	High risk	11/N/A	33.3	6/5	Clinical characteristics	Clinical characteristics	NA

M: Male, F: Female, MSAU: Multiple sclerosis-associated uveitis, EDSS: Expanded Disability Status Scale

**Fig 2:**
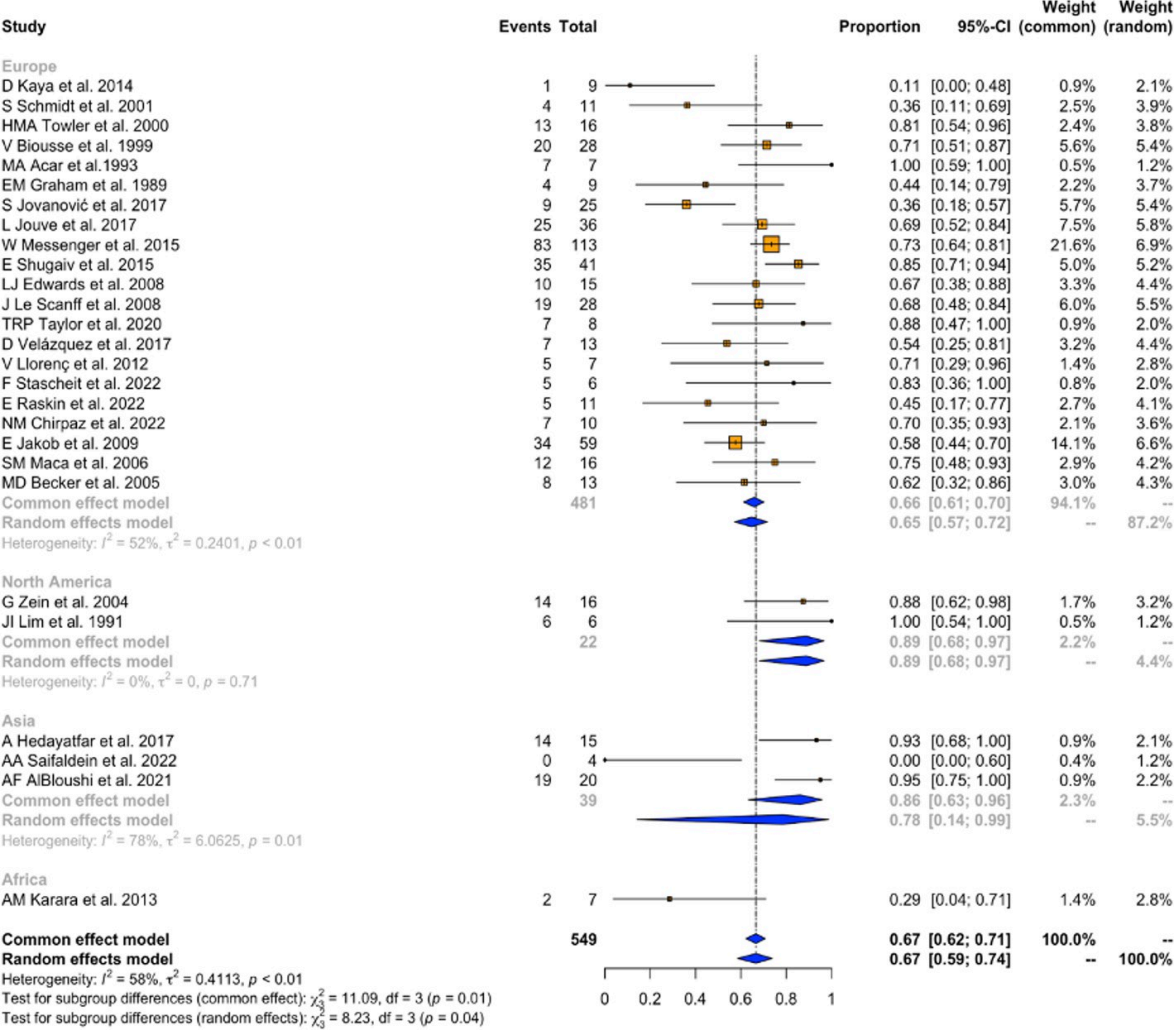
Female gender subgroup-analysis.

Of the 28 studies providing gender information for 738 patients, 67% (95% CI [59%-73%]) were female. Subgroup analysis by continent revealed a consistent female predominance across different regions, ranging from 65% (95% CI [57%-72%]) in Europe to 89% (95% CI [68%-97%]) in North America. Notably, Africa was the only continent where female predominance was not observed, with a proportion of 29% (95% CI [4%-71%]). However, this observation was based on data from only one study. The subgroup analysis exhibited moderate heterogeneity (I2 = 58%, Q = 8.23) **(Fig 2).**

### Uveitis characteristics

Twenty articles involving 629 patients documented that uveitis was presented before MS in 38% (95% CI [30%-48%]) of cases. The mean age of uveitis onset, reported in 21 articles, was 35.7 ± 8.3 years. The most frequent age stratum at presentation was 41–50 years, representing 44% (95% CI [3%-96%]; 19/39 patients), followed by 11–20 years at 32% (95% CI [19%-48%]; 28/83 patients) **(Table 2).**

**Table 2:** Uveitis characteristics and complications.

Category	No. of Studies	No. of Patients	No. eyes	Pooled prevalence 95% CI	p-value*	I2	Sensitivity Analysis	Selected Model
**Patients who first presented uveitis**	20	629		38 (30–48)	**<0.01**	77%	Negative	Random effects model
**Age presentation in years**								
1–10	3	44		28 (16–45)	0.40	0%	Negative	Random effects model
11–20	5	83		32 (19–48)	0.19	35%	Negative	Random effects model
21–30	4	40		29 (10–60)	0.05	62%	Negative	Random effects model
31–40	3	40		18 (9–33)	0.76	0%	Negative	Random effects model
41–50	3	39		44 (3–96)	**<0.01**	83%	Negative	Random effects model
51–60	2	22		16 (3–59)	0.13	55%	Negative	Random effects model
>60	No data	-	-	-	-	-	-	-
**Uveitis laterality**								
Bilateral	23	452		77 (69–83)	**<0.01**	51%	Negative	Random effects model
From unilateral to bilateral	4	144		42 (10–82)	<0.01	78%	Negative	Random effects model
Unilateral	18	483		22(14–33)	**<0.01**	78%	Negative	Random effects model
**Uveitis location**								
Intermediate	22	530		68 (49–82)	**<0.01**	82%	Negative	Random effects model
Anterior-Intermediate	3	36		30 (2–89)	**<0.01**	78%	Negative	Random effects model
Anterior	16	387		28 (17–42)	**<0.01**	74%	Negative	Random effects model
Panuveitis	11	317		26 (16–41)	**<0.01**	75%	Negative	Random effects model
Posterior	10	319		16 (8–31)	**<0.01**	83%	Negative	Random effects model
**Course**								
Recurrent	7	214		63 (38–83)	**<0.01**	92%	Negative	Random effects model
Chronic	12	294		57 (37–75)	**<0.01**	78%	Negative	Random effects model
Acute	6	190		11 (5–20)	0.20	32%	Negative	Random effects model
**Biomicroscopic findings**								
Keratic precipitates	9		186	58 (31–81)	**<0.01**	82%	Negative	Random effects model
Non-Granulomatous	5	-	52	55 (41–69)	0.31	17%	Negative	Random effect model
Granulomatous	14	-	300	49 (30–68)	**<0.01**	84%	Negative	Random effects model
Snowballs	6	-	209	35 (14–65)	**<0.01**	87%	Negative	Random effects model
**Complications**								
Posterior synechiae	6		127	62 (44–77)	**<0.01**	70%	Negative	Random effects model
Cataract	8		230	46 (32–61)	**<0.01**	72%	Negative	Random effects model
Macular compromise	4		95	45 (20–73)	**<0.01**	84%	Negative	Random effects model
Decreased vision	5		90	42 (19–70)	**<0.01**	74%	Negative	Random effects model
Retinal vascular sheathing	3		107	37 (7–81)	**<0.01**	94%	Negative	Random effects model
Periphlebitis	5		135	35 (21–52)	**0.02**	64%	Negative	Random effects model
Cystoid macular edema	10		232	26 (15–40)	**<0.01**	69%	Negative	Random effects model
Retinal detachment	3		37	18 (2–72)	**0.02**	75%	Negative	Random effects model
Epiretinal membrane	6		139	14 (9–21)	0.48	0%	Negative	Random effect model
Iris nodules	3		85	14 (8–23)	0.37	0%	Negative	Random effect model
Glaucoma	8		250	11 (5–20)	**0.03**	54%	Negative	Random effects model
Vitreous hemorrhage	3		62	10 (2–43)	**0.03**	72%	Negative	Random effects model

*: Chi-square test

**Fig 3:**
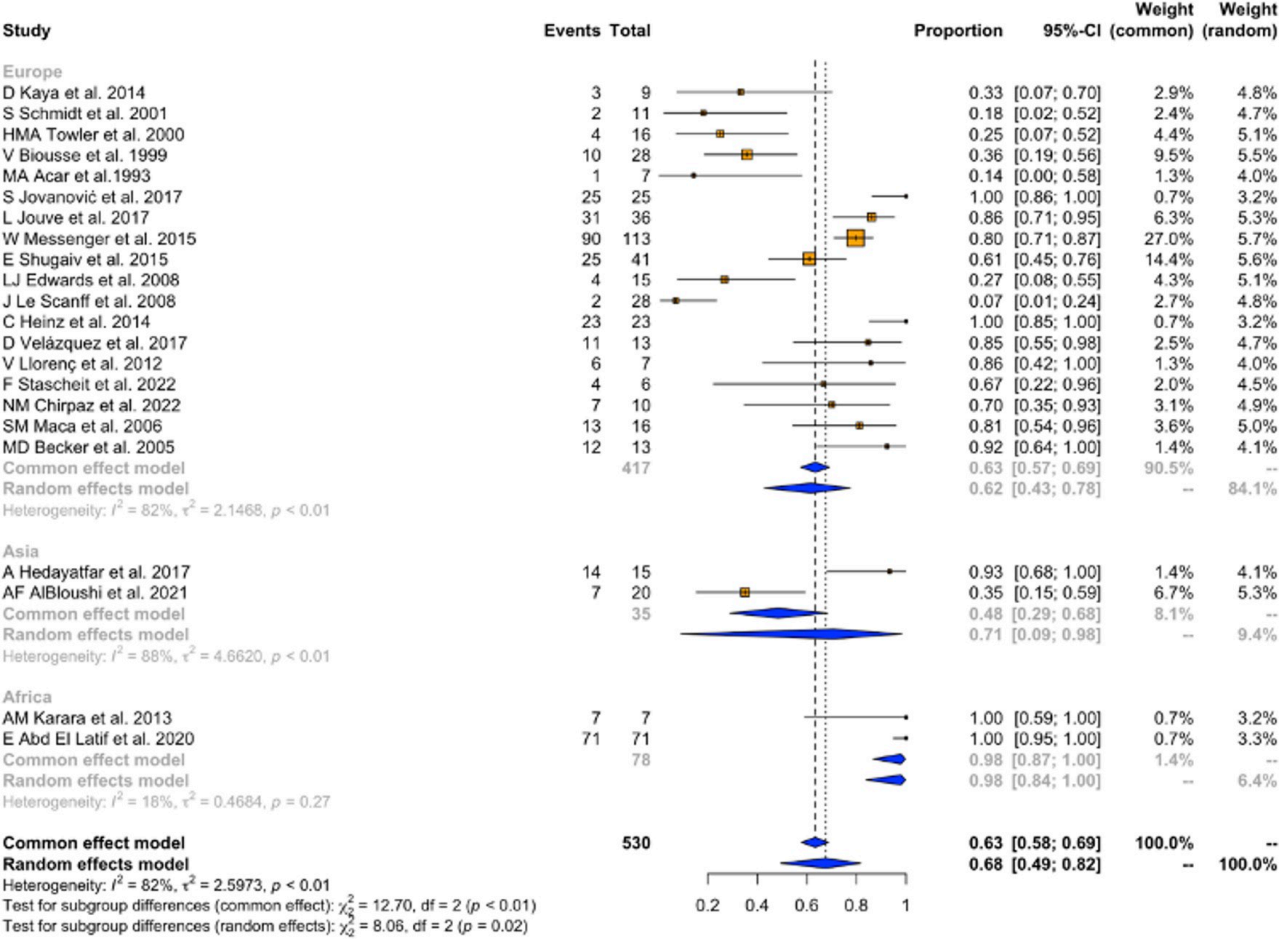
Intermediate uveitis subgroup-analysis.

Among 452 patients from 23 studies reporting laterality, 77% (95% CI [69%-83%]) presented with bilateral uveitis. Regarding anatomical location, IU was identified as the most common form, observed in 68% (95% CI [49%-82%]) of cases across 22 studies involving 530 patients **(Table 2).** The predominance of IU was evident in a subgroup analysis across geographic regions. Africa had the highest proportion of IU at 98% (95% CI [84%-100%]), followed by Asia at 71% (95% CI [9%-98%]). Notably, a high level of heterogeneity was observed in this subgroup analysis (I2 = 82%, p<0.01) **(Fig 3)**.

In 14 articles involving 300 patients, granulomatous uveitis accounted for 49% (95% CI [30%-68%]) of the studied cases. Regarding its course, uveitis was described as acute in 11% (95% CI [5%-20%]) of cases across six articles involving 190 patients, recurrent in 63% (95% CI [38%-83%]) of cases across seven articles involving 214 patients; and chronic in 57% (95% CI [37%-75%]) of cases across twelve articles involving 294 patients. The onset and duration could not be meta-analyzed due to insufficient data.

Regarding biomicroscopic findings, snowballs were documented in 35% (95% CI [14%-65%]), while snowbanks were described in 7% (95% CI [3%-14%]) of cases. Keratic precipitates were identified in 58% (95% CI [31%-81%]) of the reported cases. Other biomicroscopic findings, such as retinal vasculitis, floaters, phlebitis, frosted branch angiitis, cotton wool spots, and retinal ischemia, could not be included in the meta-analysis due to insufficient data.

Complications associated with uveitis were diverse and reported across multiple studies. Posterior synechiae occurred in 62% of cases (95% CI [44%-77%]). Cataracts were documented in 46% of patients (95% CI [32%-61%]). Macular compromise was reported in 45% of cases (95% CI [20% -73%]). Notably, decreased vision was observed in 42% of cases (95% CI [19%-70%]) across five articles involving 90 eyes **(Table 2).**

### Multiple sclerosis characteristics

Nineteen articles involving 537 patients documented that MS presented before uveitis in 59% (95% CI [48%-69%]) of cases. The mean age of neurologic symptoms reported in 11 articles was 32 ± 8.3 years. The most frequent age bracket at presentation was 11–20 years, representing 67% (95% CI [42%-85%]; 28/43 patients), followed by 0–10 years at 65% (95% CI [35%-64%]; 35/71 patients) **(Table 2).**

In terms of MS phenotype, most patients, 74% (95% CI [64%-82%]) from 134 patients across eight articles, reported a RRMS phenotype. In contrast, a smaller proportion, 9% (95% CI [3%-23%]), were classified as PPMS in three articles involving 54 patients. SPMS was documented in 24% (95% CI [18%-33%]) of cases across eight articles encompassing 125 patients.

Regarding imaging findings, supratentorial lesions were reported in 95% (95% CI [70%-99%]) of cases in two articles involving 17 patients. In comparison, spinal cord lesions were identified in 39% (95% CI [16%-68%]) of patients across two articles involving 29 patients **(Table 3).**

**Table 3:** Multiple sclerosis characteristics.

Category	No. of Studies	No. of patients	No. eyes	Pooled prevalence 95% CI	p-value*	I2	Sensitivity Analysis	Selected Model
**Neurologic signs prior to uveitis**	19	537		59 (48–69)	**<0.01**	82	Negative	Random effects model
**Age at presentation, years**								
0–10	3	71		50 (35–65)	0.19	41	Negative	Random effect model
11–20	2	43		67 (42–85)	0.14	53	Negative	Random effects model
21–30	8	99		32 (17–52)	**<0.01**	62	Negative	Random effects model
31–40	5	55		28 (18–42)	0.75	0	Negative	Random effect model
41–50	6	62		20 (12–32)	0.96	0	Negative	Random effect model
51–60	-	-		-	-	-	-	-
> 60	2	23		9 (2–31)	0.54	0	Negative	Random effect model
**Type of MS**								
Relapsing-remitting	8	134		74 (64–82)	**0.09**	43	Negative	Random effect model
Secondary progressive	8	125		24 (18–33)	0.97	0	Negative	Random effect model
Primary progressive	3	54		9 (3–23)	0.35	5	Negative	Random effect model
**Imaging findings**								
Supratentorial lesions	2	17		95 (70–99)	0.78	0	Negative	Random effect model
Spinal cord lesions	2	29		39 (16–68)	0.12	59	Negative	Random effects model

MS: Multiple sclerosis

*: Chi-square test

### Treatment characteristics

Treatment approaches for MS were recorded in 50 articles. Among these, Fingolimod, described in two articles covering 196 patients, represented a notably high percentage of 96% (95% CI [17%-100%]). Interferon beta treatment, reported in 11 articles involving 325 patients, accounted for the second highest frequency at 43% (95% CI [24%-65%]) of the cases.

Regarding uveitis, mycophenolate mofetil, found in four articles involving 51 patients, had the highest frequency at 48% (95% CI [11%-87%]). Azathioprine, reported in 8 articles comprising 145 patients, accounted for 21% (95% CI [9%-41%]) of the cases, while methotrexate use was documented in three articles encompassing 36 patients, comprising 9% (95% CI [3%-24%]) of the cases **(Table 4).**

**Table 4:** Treatment for multiple sclerosis-associated uveitis.

Treatment for neurological manifestations								
Category	No. of Studies	No. of patients	No. eyes	Pooled prevalence 95% CI	p-value*	I2	Sensitivity Analysis	Selected Model
Treatment with Fingolimod	2	196		96 (17–100)	<0.01	89%	Negative	Random effects model
Treatment with Beta Interferon	11	325		43 (24–65)	<0.01	85%	Negative	Random effects model
Treatment with Glatiramer	5	183		11 (7–16)	0.91	0%	Negative	Random effects model
Treatment with Natalizumab	2	149		4 (1–20)	0.42	0%	Negative	Random effects model
**Treatment for uveitis**								
**Category**	**No. of Studies**	**No. of patients**	**No. eyes**	**Pooled prevalence 95% CI**	p-value*	**I2**	**Sensitivity Analysis**	**Selected Model**
Treatment with Mycophenolate	4	51		48 (11–87)	0.02	71%%	Negative	Random effects model
Treatment with Tacrolimus	2	22		38 (4–90)	0.01	84%	Negative	Random effects model
Treatment with Azathioprine	8	145		21(9–41)	<0.01	73%	Negative	Random effects model
Treatment with Ciclosporin	4	44		17 (8–34)	0.27	23%	Negative	Random effects model
Treatment with Methotrexate	3	36		9 (3–24)	0.82	0%	Negative	Random effects model

*: Chi-square test

## Discussion

**Fig 4:**
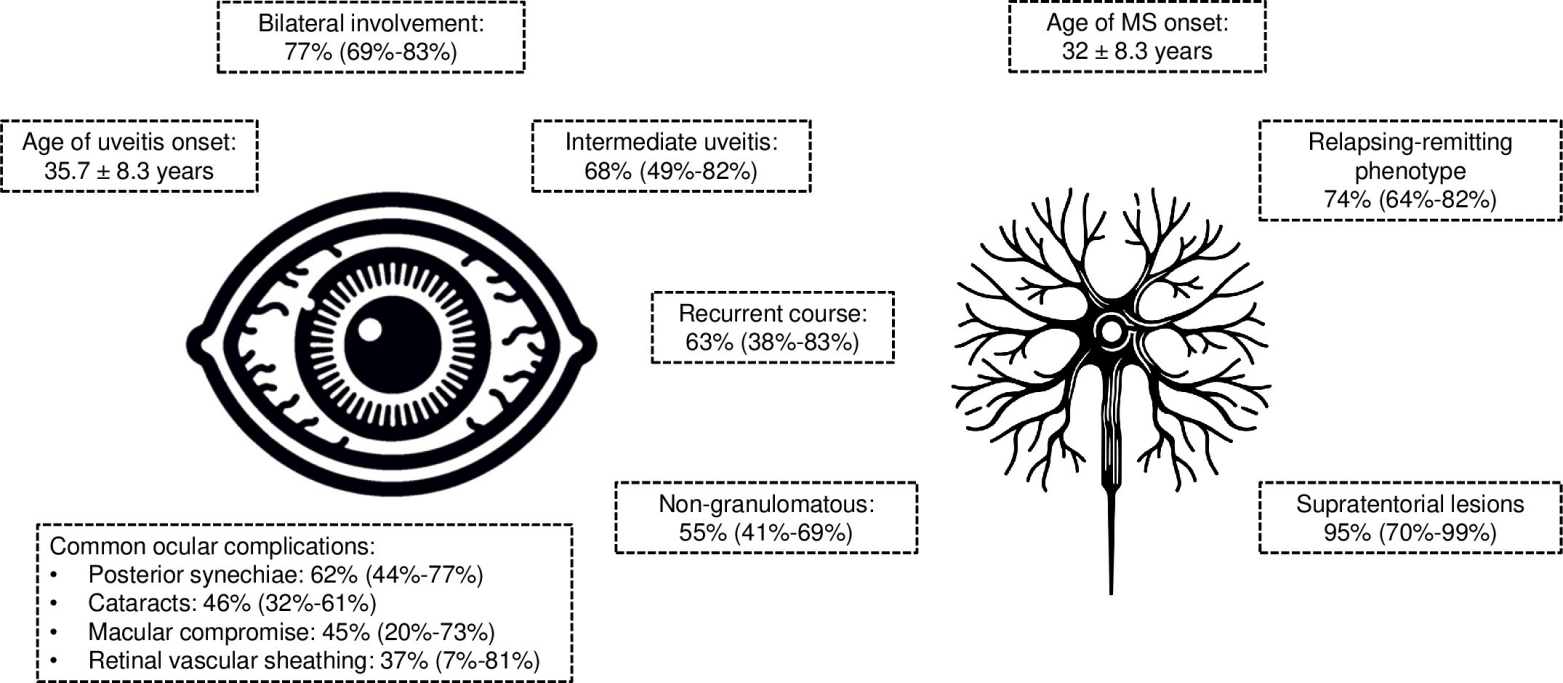
Clinical picture of patients with multiple sclerosis-associated uveitis.

This systematic review evaluated the characteristics of uveitis, ocular complications, and MS phenotype of patients with MSAU **(Fig 4).** These findings offer valuable insights into the clinical profile of these patients, aiding ophthalmologists and neurologists in making prompt and accurate diagnoses, which are usually complex.

For instance, uveitis has been identified as an initial symptom in 23% of MS patients [43]. Timely neurological monitoring following the first episode of uveitis could facilitate early diagnosis and appropriate treatment [12]. In addition, patients with standalone MS may require a distinct treatment approach compared to those suffering from MSAU [7]. Moreover, it’s essential to differentiate between the various eye-related conditions in MS. Patients commonly exhibit optic neuritis and abnormalities in oculomotor function, each requiring distinct treatment approaches [4, 56].

With this in mind, we found a mean age of presentation of 38.2 ± 12.1 years, with a notable female predominance (68%). This observation aligns with the findings of a retrospective study conducted by Messenger et al., which included 113 individuals with MSAU, of which 83 (73.5%) were female and 30 (26,5%) were male [43]. This female preponderance has been linked to common autoinflammatory pathways between both diseases and their relationship with sex hormones, a concept known as autoimmune tautology [57, 58].

Although there was a significant degree of heterogeneity among studies in the subgroup analyses by continent, as expected due to study designs, a consistent pattern of female predominance **(Fig 1)** was evident when compared to male. Specifically, 65% of the included patients in Europe were female; this proportion increased to 89% in North America. It is worth mentioning that although genetic factors may influence sex predominance and studies conducted in North America did not exhibit significant heterogeneity (I2: 0%, p = 0.71), this observation was based on data from only two studies. Additionally, only one study has been conducted in Africa. This underscores the need for more comprehensive studies to elucidate the relationship between sex predominance and geographical factors.

### Uveitis characteristics

Intermediate uveitis has been described as the most common anatomical localization of uveitis in patients with MS [43, 59]. Similarly, in our study, IU accounted for 68% (95% CI: 49–82) of the cases, followed by anterior and IU combined, which was observed in 30% (95% CI: 2–89) of the cases. Notably, uveitis can manifest in other ocular locations, such as posterior uveitis, affecting 16% (95% CI: 8–31) of patients, or panuveitis, affecting 26% (95% CI: 16–41) of patients.

The localization of uveitis can vary depending on the genetic and immunological pathways of the host. Specific HLA Human Leukocyte Antigen (HLA) has been associated with specific uveitis localizations. One well-known example is the HLA-B27, which is linked to anterior uveitis in patients with spondylarthritis [60]. In the case of IU, Lindner et al. identified a gene polymorphism in IL2RA (rs2104286) that increases susceptibility to IU risk (84.5% vs. 15.5%) [61]. This polymorphism has been linked to reduced expression of CD25 on CD4 naive T cells and CD14CD16 monocytes, potentially decreasing the activation of naive CD4 T cells under pro-inflammatory conditions [61, 62]. Exploring genetic therapies to address intraocular inflammation could open new possibilities for treating refractory MSAU. The eye is considered a promising target due to its small, compartmentalized shape, which might require a relatively small number of vectors/gene copies, and its special immune features that can favor viral-mediated gene therapy [63].

The subgroup analysis consistently observed IU predominance by continents **(Fig 3).** In Europe, intermediate uveitis demonstrated a pooled prevalence of 62%, which notably increased to 98% in Africa. While genetic factors might contribute [64, 65] to the variations between groups, this can also be attributed to the relatively limited number of studies conducted outside Europe and the substantial heterogeneity observed among subgroups (82%).

In our study, the mean age of the first uveitis episode was 35.7 ± 8.3 years, while the mean age at MS onset was 32 ± 8.35 years. Furthermore, the prevalence of patients developing MS before their first uveitis episode was 59% higher than in the cases where uveitis preceded MS (38%). It is worth noting that there is contradictory information in the literature on this topic, with some studies reporting uveitis preceding MS and other studies indicating neurological symptoms occurring before ocular involvement [43, 66] or simultaneously [43]. Regardless of which precedes, this finding underscores the critical importance of communication and collaboration between ophthalmologists and neurologists in managing uveitis and MS patients.

In terms of the course, chronic uveitis (57% [95% CI: 37%-75%]) and recurrent uveitis 63% [95% CI: 38%-83%]) were the most frequently observed, often affecting both eyes (77% [95% CI: 69%-83%]). These findings have been previously reported in other studies and warrant special attention due to their association with severe and challenging-to-treat inflammation [67]. It is important to note that chronic uveitis is identified as a risk factor for ocular complications (OR: 1.78, p = 0.002) [67]. Indeed, our study revealed a higher overall prevalence of complications, including posterior synechiae (62%), cataracts (46%), macular compromise (45%), and vascular sheathing (37%). Studies have indicated that the severity of uveitis tends to be lower in patients who receive systemic treatment [46]. Hence, individuals with MS should undergo regular evaluations to tailor treatment and effectively manage ocular or systemic inflammation.

### Multiple sclerosis characteristics

Concerning the clinical phenotype of MS, RRMS exhibited the highest pooled prevalence at 74% (95% CI 64–82), followed by SPMS at 24% (95% CI 18–33). Notably, RRMS has consistently been reported as the predominant course in other studies, followed by SPMS [33, 68]. The RRMS predominance in this study can also explain the minimal and moderate disability reported in the EDSS of 2.9 ± 0.6 [69]. PPMS is considered relatively rare (with a prevalence accounting for 7% to 57%) [70, 71].

### Strength and limitations

This is the first meta-analysis to comprehensively describe the uveitis characteristics and phenotype of MS in patients with MSAU. This study enables the creation of a large sample that would not be feasible through other means, offering insights into the clinical picture of patients with MSAU for ophthalmologists and neurologists.

This study has some limitations. First, it encompassed research conducted over a broad period, during which multiple criteria for diagnosing MS evolved, influenced by clinical knowledge and the dissemination of new technologies such as magnetic resonance imaging. Nevertheless, all studies were included if the diagnosis of MS was based on the most relevant criteria available at the time, from the Poser criteria in 1983 to the McDonald criteria in 2017.

Second, most of the included studies were from Europe, with only a few originating from Asia and Africa. For South America, no studies were identified. Nonetheless, the data from the few studies in these regions indicated that patients shared similar characteristics with Europeans, including female predominance and IU localization. Further research is needed to clarify the clinical presentations of these regions.

Thirdly, it’s crucial to emphasize that the quality of existing evidence is limited, a common challenge in rare diseases like MSAU. Most literature primarily consists of cross-sectional studies, underscoring the need for prospective cohort studies and clinical trials. Such endeavors are essential for improving the quality of evidence and pinpointing characteristics with greater precision, such as the temporal relationship between the onset of uveitis and MS.

Fourth, the considerable heterogeneity identified in some of the pooled prevalences is a limitation that was anticipated. However, it is worth noting that in the subgroup analysis of variables, such as intermediate localization, the I2 values were lower, indicating more favorable levels of heterogeneity. Lastly, our analysis did not delve into treatment strategies with specific outcomes such as visual acuity or uveitis relapses. Further studies could provide valuable insights into managing MSAU cases.

Finally, we may have missed some important reports due to using a cohort threshold of 10 patients or eyes. The decision to exclude studies with fewer than ten observations was based on methodological considerations like previous studies [72–75]. In meta-analyses using random effects models, the weights assigned to individual studies are similar across observations. This means that an event occurring in 1 out of 3 cases (33%) can disproportionately influence the results compared to an event in 1 out of 10 cases (10%). Such discrepancies can lead to bias in the measurement of central tendency, as one outlier can significantly skew the overall results [72].

## Conclusion

This systematic review summarizes and validates uveitis characteristics and MS phenotype of patients with MSAU, which is heterogeneous across studies. MSAU primarily affects young adult females with bilateral involvement. Intermediate uveitis is the most prevalent localization and can occur simultaneously, before or after the diagnosis of MS. Ocular complications, including cataracts, macular compromise, and vascular retinal alterations, may develop. Regarding MS phenotype, RRMS is more frequently observed in these patients, often accompanied by supratentorial lesions as revealed by MRI scans. We advocate for interdisciplinary collaboration among specialists when treating patients with these clinical features to expedite diagnosis and minimize potential complications.

## Supporting information

S1 Checklist PRISMA checklist. (DOCX)

S1 File Search strategy. (DOCX)

S2 File Risk of bias. (DOCX)

S3 File Sensitivity analysis. (DOCX)

S1 Raw data (XLSX)

## References

[1] Walton C, King R, Rechtman L, Kaye W, Leray E, Marrie RA, et al. Rising prevalence of multiple sclerosis worldwide: Insights from the Atlas of MS, third edition. Mult Scler. 2020;26: 1816–1821. doi: 10.1177/1352458520970841 33174475 PMC7720355

[2] Cunningham ET, Pavesio CE, Goldstein DA, Forooghian F, Zierhut M. Multiple Sclerosis-Associated Uveitis. Ocular Immunology and Inflammation. 2017;25: 299–301. doi: 10.1080/09273948.2017.1334469 28696171

[3] McGinley MP, Goldschmidt CH, Rae-Grant AD. Diagnosis and Treatment of Multiple Sclerosis: A Review. JAMA. 2021;325: 765. doi: 10.1001/jama.2020.26858 33620411

[4] Toosy AT, Mason DF, Miller DH. Optic neuritis. The Lancet Neurology. 2014;13: 83–99. doi: 10.1016/S1474-4422(13)70259-X 24331795

[5] Zhao R-Z, Zhang G-X, Zhang W-T, Yu W-J, Du L, Toledo MC, et al. Ocular manifestations of multiple sclerosis in patients from three countries: A Web-based survey. European Journal of Ophthalmology. 2022;32: 2975–2981. doi: 10.1177/11206721211069457 34939452

[6] Optic Neuritis Study Group*. The 5-year risk of MS after optic neuritis: Experience of the Optic Neuritis Treatment Trial. Neurology. 1997;49: 1404–1413. doi: 10.1212/WNL.49.5.1404 9371930

[7] Casselman P, Cassiman C, Casteels I, Schauwvlieghe P. Insights into multiple sclerosis‐associated uveitis: a scoping review. Acta Ophthalmologica. 2021;99: 592–603. doi: 10.1111/aos.14697 33326162

[8] Gordon LK, Goldstein DA. Gender and Uveitis in Patients with Multiple Sclerosis. Journal of Ophthalmology. 2014;2014: 1–5. doi: 10.1155/2014/565262 24891944 PMC4033526

[9] Olsen TG, Frederiksen J. The association between multiple sclerosis and uveitis. Survey of Ophthalmology. 2017;62: 89–95. doi: 10.1016/j.survophthal.2016.07.002 27491475

[10] Marrie RA, Cutter G, Tyry T. Substantial adverse association of visual and vascular comorbidities on visual disability in multiple sclerosis. Mult Scler. 2011;17: 1464–1471. doi: 10.1177/1352458511414041 21844067

[11] Kaya D, Kaya M, Özakbaş S, İdiman E. Uveitis associated with multiple sclerosis: complications and visual prognosis. Int J Ophthalmol. 2014;7: 1010–1013. doi: 10.3980/j.issn.2222-3959.2014.06.18 25540756 PMC4270967

[12] Zein G, Berta A, Foster S. Multiple sclerosis-associated uveitis. Ocular Immunology and Inflammation. 2004;12: 137–142. doi: 10.1080/09273940490895344 15512983

[13] Fitoussi R, Gascon P, Denis D, Mathis T, Tieulé N, Schneider-Rouhaud C, et al. Epidemiological, Clinical, and Therapeutic Profile of Uveitis in Multiple Sclerosis: A Multicenter Study. Ocular Immunology and Inflammation. 2024; 1–5. doi: 10.1080/09273948.2024.2337839 38602890

[14] Murad MH, Sultan S, Haffar S, Bazerbachi F. Methodological quality and synthesis of case series and case reports. BMJ EBM. 2018;23: 60–63. doi: 10.1136/bmjebm-2017-110853 29420178 PMC6234235

[15] Google Translate. Available: https://translate.google.com/?hl=es&sl=en&tl=es&op=translate

[16] Thompson AJ, Banwell BL, Barkhof F, Carroll WM, Coetzee T, Comi G, et al. Diagnosis of multiple sclerosis: 2017 revisions of the McDonald criteria. The Lancet Neurology. 2018;17: 162–173. doi: 10.1016/S1474-4422(17)30470-2 29275977

[17] Poser CM, Paty DW, Scheinberg L, McDonald WI, Davis FA, Ebers GC, et al. New diagnostic criteria for multiple sclerosis: Guidelines for research protocols. Annals of Neurology. 1983;13: 227–231. doi: 10.1002/ana.410130302 6847134

[18] CLARITY Group. Tool to Assess Risk of Bias in Cohort Studies. Available: https://www.distillersr.com/resources/methodological-resources/tool-to-assess-risk-of-bias-in-cohort-studies-distillersr

[19] Hoy D, Brooks P, Woolf A, Blyth F, March L, Bain C, et al. Assessing risk of bias in prevalence studies: modification of an existing tool and evidence of interrater agreement. Journal of Clinical Epidemiology. 2012;65: 934–939. doi: 10.1016/j.jclinepi.2011.11.014 22742910

[20] CLARITY Group. Tool to Assess Risk of Bias in Case Control Studies. Available: https://www.distillersr.com/resources/methodological-resources/tool-to-assess-risk-of-bias-in-case-control-studies-distillersr.

[21] Risk of bias tools—robvis (visualization tool). [cited 7 Dec 2023]. Available: https://www.riskofbias.info/welcome/robvis-visualization-tool

[22] Deeks J, Higgins JP, Altman D. Chapter 10: Analysing data and undertaking meta-analyses. Available: https://training.cochrane.org/handbook/current/chapter-10#section-10-10

[23] Graham EM, Francis DA, Sanders MD, Rudge P. Ocular inflammatory changes in established multiple sclerosis. Journal of Neurology, Neurosurgery & Psychiatry. 1989;52: 1360–1363. doi: 10.1136/jnnp.52.12.1360 2614430 PMC1031592

[24] Lim JI, Tessler HH, Goodwin JA. Anterior Granulomatous Uveitis in Patients with Multiple Sclerosis. Ophthalmology. 1991;98: 142–145. doi: 10.1016/s0161-6420(91)32324-8 2008270

[25] Acar MA, Birch MK, Abbott R, Rosenthal AR. Chronic granulomatous anterior uveitis associated with multiple sclerosis. Graefe’s Arch Clin Exp Ophthalmol. 1993;231: 166–168. doi: 10.1007/BF00920941 8462890

[26] Biousse V, Trichet C, Bloch-Michel E, Roullet E. Multiple sclerosis associated with uveitis in two large clinic-based series. Neurology. 1999;52: 179–179. doi: 10.1212/wnl.52.1.179 9921871

[27] Towler HM, Lightman S. Symptomatic intraocular inflammation in multiple sclerosis. Clinical Exper Ophthalmology. 2000;28: 97–102. doi: 10.1046/j.1442-9071.2000.00270.x 10933771

[28] Schmidt S, Wessels L, Augustin A, Klockgether T. Patients with Multiple Sclerosis and concomitant uveitis/periphlebitis retinae are not distinct from those without intraocular inflammation. Journal of the Neurological Sciences. 2001;187: 49–53. doi: 10.1016/s0022-510x(01)00520-2 11440744

[29] Prieto JF, Dios E, Gutierrez JM, Mayo A, Calonge M, Herreras JM. Pars planitis: epidemiology, treatment, and association with multiple sclerosis. Ocular Immunology and Inflammation. 2001;9: 93–102. doi: 10.1076/ocii.9.2.93.3975 11449325

[30] Edwards LJ, Constantinescu CS. A prospective study of conditions associated with multiple sclerosis in a cohort of 658 consecutive outpatients attending a multiple sclerosis clinic. Mult Scler. 2004;10: 575–581. doi: 10.1191/1352458504ms1087oa 15471376

[31] Becker MD. Interferon as a treatment for uveitis associated with multiple sclerosis. British Journal of Ophthalmology. 2005;89: 1254–1257. doi: 10.1136/bjo.2004.061119 16170111 PMC1772902

[32] Maca SM, Scharitzer M, Barisani-Asenbauer T. Uveitis and neurologic diseases: an often overlooked relationship. Wien Klin Wochenschr. 2006;118: 273–279. doi: 10.1007/s00508-006-0601-6 16810485

[33] Le Scanff J, Sève P, Renoux C, Broussolle C, Confavreux C, Vukusic S. Uveitis associated with multiple sclerosis. Mult Scler. 2008;14: 415–417. doi: 10.1177/1352458507083444 18208897

[34] Edwards LJ, Dua H, Constantinescu CS. Symptomatic Uveitis and Multiple Sclerosis. Neuro-Ophthalmology. 2008;32: 49–54. doi: 10.1080/01658100701551419

[35] Jakob E, Reuland MS, Mackensen F, Harsch N, Fleckenstein M, Lorenz H-M, et al. Uveitis Subtypes in a German Interdisciplinary Uveitis Center—Analysis of 1916 Patients. J Rheumatol. 2009;36: 127–136. doi: 10.3899/jrheum.080102 19132784

[36] Paovic J, Paovic P, Vukosavljevic M. Clinical and immunological features of retinal vasculitis in systemic diseases. VSP. 2009;66: 961–965. doi: 10.2298/vsp0912961p 20095515

[37] Langer-Gould A, Albers K, Van Den Eeden S, Nelson L. Autoimmune diseases prior to the diagnosis of multiple sclerosis: a population-based case-control study. Mult Scler. 2010;16: 855–861. doi: 10.1177/1352458510369146 20463037

[38] Kanavi MR, Soheilian M, Naghshgar N. Confocal Scan of Keratic Precipitates in Uveitic Eyes of Various Etiologies. Cornea. 2010;29: 650–654. doi: 10.1097/ICO.0b013e3181c2967e 20458232

[39] Llorenç V, Rey A, Mesquida M, Pelegrín L, Adán A. Uveítis asociadas a enfermedad desmielinizante del sistema nervioso central. Archivos de la Sociedad Española de Oftalmología. 2012;87: 324–329. doi: 10.1016/j.oftal.2012.04.027 23021230

[40] Karara AM, Macky TA, Sharawy MH. Pattern of Uveitis in an Egyptian Population with Multiple Sclerosis: A Hospital-Based Study. Ophthalmic Res. 2013;49: 25–29. doi: 10.1159/000341735 23007229

[41] Heinz C, Schoonbrood S, Heiligenhaus A. Intermediate uveitis in children and young adults: differences in clinical course, associations and visual outcome. Br J Ophthalmol. 2014;98: 1107–1111. doi: 10.1136/bjophthalmol-2013-304589 24713505

[42] Shugaiv E, Tüzün E, Kürtüncü M, Kıyat-Atamer A, Çoban A, Akman-Demir G, et al. Uveitis as a prognostic factor in multiple sclerosis. Mult Scler. 2015;21: 105–107. doi: 10.1177/1352458514539782 24948689

[43] Messenger W, Hildebrandt L, Mackensen F, Suhler E, Becker M, Rosenbaum JT. Characterisation of uveitis in association with multiple sclerosis. Br J Ophthalmol. 2015;99: 205–209. doi: 10.1136/bjophthalmol-2014-305518 25170065

[44] Velazquez-Villoria D, Macia-Badia C, Segura-García A, Pastor Idoate S, Arcos-Algaba G, Velez-Escola L, et al. Eficacia del tratamiento inmunomodulador con interferón-β o acetato de glatirámero en las uveítis asociadas a esclerosis múltiple. Archivos de la Sociedad Española de Oftalmología. 2017;92: 273–279. doi: 10.1016/j.oftal.2016.11.018 28188020

[45] Hedayatfar A, Falavarjani KG, Soheilian M, Elmi Sadr N, Modarres M, Parvaresh MM, et al. Mycophenolate Mofetil for the Treatment of Multiple Sclerosis-associated Uveitis. Ocular Immunology and Inflammation. 2017;25: 308–314. doi: 10.1080/09273948.2016.1178302 27379567

[46] Jouve L, Benrabah R, Héron E, Bodaghi B, Le Hoang P, Touitou V. Multiple Sclerosis-related Uveitis: Does MS Treatment Affect Uveitis Course? Ocular Immunology and Inflammation. 2017;25: 302–307. doi: 10.3109/09273948.2015.1125508 26902594

[47] Jovanović S, Šarenac Vulović T, Radotić F, Tončić Z, Živković M, Petrović N. Quantitative Analysis of Uveitis Macular Edema in Multiple Sclerosis Patients Receiving Deep Posterior Sub-Tenon Triamcinolone Acetonide Injection. Ophthalmic Res. 2017;58: 1–7. doi: 10.1159/000458157 28324879

[48] Lim LL, Silva DG, Lo TC, Pimentel RS, Butzkueven H, Hall AJ. Uveitis in Patients with Multiple Sclerosis in Clinical Trials of Fingolimod. Ophthalmology. 2019;126: 438–444. doi: 10.1016/j.ophtha.2018.10.013 30315901

[49] Taylor TR, Jacobs BM, Giovannoni G, Petrushkin H, Dobson R. Prevalence and demographics of multiple sclerosis-associated uveitis: a UK biobank study. Multiple Sclerosis and Related Disorders. 2020;43: 102209. doi: 10.1016/j.msard.2020.102209 32480346

[50] Abd El Latif E, Abdelhalim AS, Montasser AS, Said MH, Shikhoun Ahmed M, Abdel Kader Fouly Galal M, et al. Pattern of Intermediate Uveitis in an Egyptian Cohort. Ocular Immunology and Inflammation. 2020;28: 524–531. doi: 10.1080/09273948.2019.1668429 31642742

[51] AlBloushi AF, Dheyab AM, Al-Swaina NF, Al-Obailan M, Daif A, Abu El-Asrar AM. Clinical findings and outcomes of uveitis associated with multiple sclerosis. European Journal of Ophthalmology. 2021;31: 482–490. doi: 10.1177/1120672120904667 32019337

[52] Saifaldein AA, AlBloushi AF, Altariqi SM, Aljarallah S, Abu El-Asrar AM. Occlusive Retinal Vasculitis in Patients with Multiple Sclerosis. Ocular Immunology and Inflammation. 2023;31: 1750–1757. doi: 10.1080/09273948.2022.2103717 35914306

[53] Chirpaz N Md, Kerever S Md, PhD, Gavoille A Md, Kodjikian L Md, PhD, Bernier R Md, Gerfaud-Valentin M Md, et al. Relevance of Brain MRI in Patients with Uveitis: Retrospective Cohort on 402 Patients. Ocular Immunology and Inflammation. 2022;30: 1109–1115. doi: 10.1080/09273948.2020.1870145 33826481

[54] Stascheit F, Rübsam A, Otto C, Meisel A, Ruprecht K, Pleyer U. Anti‐CD20 therapy for multiple sclerosis‐associated uveitis: A case series. Euro J of Neurology. 2022;29: 3028–3038. doi: 10.1111/ene.15453 35716269

[55] Raskin E, Achiron A, Zloto O, Neuman R, Vishnevskia-Dai V. Uveitis prior to clinical presentation of Multiple Sclerosis (MS) is associated with better MS prognosis. Filippi M, editor. PLoS ONE. 2022;17: e0264918. doi: 10.1371/journal.pone.0264918 35767533 PMC9242474

[56] Tilikete C, Jasse L, Vukusic S, Durand‐Dubief F, Vardanian C, Pélisson D, et al. Persistent ocular motor manifestations and related visual consequences in multiple sclerosis. Annals of the New York Academy of Sciences. 2011;1233: 327–334. doi: 10.1111/j.1749-6632.2011.06116.x 21951012

[57] Deretzi G, Kountouras J, Polyzos SA, Koutlas E, Pelidou S-H, Xeromerisiou G, et al. Polyautoimmunity in a Greek cohort of multiple sclerosis. Acta Neurol Scand. 2015;131: 225–230. doi: 10.1111/ane.12308 25270060

[58] Anaya J-M. The autoimmune tautology. Arthritis Res Ther. 2010;12: 147, ar3175. doi: 10.1186/ar3175 21092150 PMC3046506

[59] Abraham A, Nicholson L, Dick A, Rice C, Atan D. Intermediate uveitis associated with MS: Diagnosis, clinical features, pathogenic mechanisms, and recommendations for management. Neurol Neuroimmunol Neuroinflamm. 2021;8: e909. doi: 10.1212/NXI.0000000000000909 33127747 PMC7641065

[60] Linssen A, Rothova A, Valkenburg HA, Dekker-Saeys AJ, Luyendijk L, Kijlstra A, et al. The lifetime cumulative incidence of acute anterior uveitis in a normal population and its relation to ankylosing spondylitis and histocompatibility antigen HLA-B27. Invest Ophthalmol Vis Sci. 1991;32: 2568–2578. 1869411

[61] Lindner E, Weger M, Steinwender G, Griesbacher A, Posch U, Ulrich S, et al. IL2RA Gene Polymorphism rs2104286 A>G Seen in Multiple Sclerosis Is Associated with Intermediate Uveitis: Possible Parallel Pathways? Invest Ophthalmol Vis Sci. 2011;52: 8295. doi: 10.1167/iovs.11-8163 21911588

[62] Dendrou CA, Plagnol V, Fung E, Yang JHM, Downes K, Cooper JD, et al. Cell-specific protein phenotypes for the autoimmune locus IL2RA using a genotype-selectable human bioresource. Nat Genet. 2009;41: 1011–1015. doi: 10.1038/ng.434 19701192 PMC2749506

[63] Ghoraba HH, Akhavanrezayat A, Karaca I, Yavari N, Lajevardi S, Hwang J, et al. Ocular Gene Therapy: A Literature Review with Special Focus on Immune and Inflammatory Responses. OPTH. 2022;Volume 16: 1753–1771. doi: 10.2147/OPTH.S364200 35685379 PMC9173725

[64] Khankhanian P, Matsushita T, Madireddy L, Lizée A, Din L, Moré JM, et al. Genetic contribution to multiple sclerosis risk among Ashkenazi Jews. BMC Med Genet. 2015;16: 55. doi: 10.1186/s12881-015-0201-2 26212423 PMC4557862

[65] Marrosu M. DRB1-DQA1-DQB1 loci and multiple sclerosis predisposition in the Sardinian population. Human Molecular Genetics. 1998;7: 1235–1237. doi: 10.1093/hmg/7.8.1235 9668164

[66] Marrie RA, Reider N, Stuve O, Trojano M, Sorensen PS, Cutter GR, et al. The incidence and prevalence of comorbid gastrointestinal, musculoskeletal, ocular, pulmonary, and renal disorders in multiple sclerosis: A systematic review. Mult Scler. 2015;21: 332–341. doi: 10.1177/1352458514564488 25538150 PMC4429162

[67] Prieto-del-Cura M, González-Guijarro JJ. Risk factors for ocular complications in adult patients with uveitis. European Journal of Ophthalmology. 2020;30: 1381–1389. doi: 10.1177/1120672119899379 31902244

[68] Perrone V, Veronesi C, Giacomini E, Citraro R, Dell’Orco S, Lena F, et al. The Epidemiology, Treatment Patterns and Economic Burden of Different Phenotypes of Multiple Sclerosis in Italy: Relapsing-Remitting Multiple Sclerosis and Secondary Progressive Multiple Sclerosis. CLEP. 2022;Volume 14: 1327–1337. doi: 10.2147/CLEP.S376005 36387930 PMC9648183

[69] Leray E, Moreau T, Fromont A, Edan G. Epidemiology of multiple sclerosis. Revue Neurologique. 2016;172: 3–13. doi: 10.1016/j.neurol.2015.10.006 26718593

[70] Amato MP, Goretti B. Cognitive Impairment in Multiple Sclerosis. Translational Neuroimmunology in Multiple Sclerosis. Elsevier; 2016. pp. 365–384. doi: 10.1016/B978-0-12-801914-6.00027-1

[71] Abdelhak A, Weber MS, Tumani H. Primary Progressive Multiple Sclerosis: Putting Together the Puzzle. Front Neurol. 2017;8: 234. doi: 10.3389/fneur.2017.00234 28620346 PMC5449443

[72] Peterson AM, Takiya L, Finley R. Meta-analysis of trials of interventions to improve medication adherence. American Journal of Health-System Pharmacy. 2003;60: 657–665. doi: 10.1093/ajhp/60.7.657 12701547

[73] Hattle M, Burke DL, Trikalinos T, Schmid CH, Chen Y, Jackson D, et al. Multivariate meta-analysis of multiple outcomes: characteristics and predictors of borrowing of strength from Cochrane reviews. Syst Rev. 2022;11: 149. doi: 10.1186/s13643-022-01999-0 35883187 PMC9316363

[74] Putera I, Ridwan AS, Dewi M, Cifuentes-González C, Rojas-Carabali W, Sitompul R, et al. Antiviral treatment for acute retinal necrosis: A systematic review and meta-analysis. Survey of Ophthalmology. 2024;69: 67–84. doi: 10.1016/j.survophthal.2023.09.004 37774799

[75] Mejía-Salgado G, Muñoz-Vargas PT, Cifuentes-González C, Flórez-Esparza G, Paquentín-Jiménez R, Castro-Monreal MÁ, et al. Quantitative changes in the corneal endothelium and central corneal thickness during anterior chamber inflammation: A systematic review and meta-analysis. Grzybowski A, editor. PLoS ONE. 2024;19: e0296784. doi: 10.1371/journal.pone.0296784 38181008 PMC10769021

