# Supplementary material for: Uveitis characteristics and multiple sclerosis phenotype of patients with multiple sclerosis-associated uveitis: A systematic review and meta-analysis
Source: PLoS One. 2024 Oct 25;19(10):e0307455. doi: 10.1371/journal.pone.0307455 (PMC11508149; doi:10.1371/journal.pone.0307455)
Supplement: S1 File — (DOCX) [file pone.0307455.s002.docx]

**Supplementary information 2: Search strategy**

SEARCH STRATEGY PUBMED – DATE 25 JANUARY 2023

| TERMS | SEARCH |
| --- | --- |
| Multiple Sclerosi  Results:  96,996 | (Multiple Sclerosis[MeSH Terms]) OR (Multiple Sclerosis[Title/Abstract])) |
| Uveitis  Results:  43,431 | (((((((Iridocyclitis[MeSH Terms]) OR (Iridocyclitis[Title/Abstract])) OR (Iritis[MeSH Terms])) OR (Iritis[Title/Abstract])) OR (Uveitis[MeSH Terms])) OR (Uveitis[Title/Abstract])) OR (retinal vasculitis[MeSH Terms])) OR (retinal vasculitis[Title/Abstract]) |
| Search straegy  Results:  569 | ("multiple sclerosis"[MeSH Terms] OR "multiple sclerosis"[Title/Abstract]) AND ("Iridocyclitis"[MeSH Terms] OR "Iridocyclitis"[Title/Abstract] OR "Iritis"[MeSH Terms] OR "Iritis"[Title/Abstract] OR "Uveitis"[MeSH Terms] OR "Uveitis"[Title/Abstract] OR "retinal vasculitis"[MeSH Terms] OR "retinal vasculitis"[Title/Abstract]) |

SEARCH STRATEGY EMBASE – DATE 25 JANUARY 2023

| TERMS | SEARCH |
| --- | --- |
| Multiple Sclerosi  Results:  171,473 | 'multiple sclerosis'/exp OR 'multiple sclerosis':ti,ab,kw |
| Uveitis  Results:  73,559 | 'uveitis'/exp OR uveitis:ti,ab,kw OR 'retina vasculitis'/exp OR 'retina vasculitis':ti,ab,kw |
| Search straegy  Results:  1,856 | ('multiple sclerosis'/exp OR 'multiple sclerosis':ti,ab,kw) AND ('uveitis'/exp OR uveitis:ti,ab,kw OR 'retina vasculitis'/exp OR 'retina vasculitis':ti,ab,kw) |

SEARCH STRATEGY Virtual Health Library (VHL) – DATE 25 JANUARY 2023

| TERMS | SEARCH |
| --- | --- |
| Multiple Sclerosi  Results:  119,881 | (Multiple Sclerosis) |
| Uveitis  Results:  28.738 | (Uveitis) OR (Retinal Vasculitis) |
| Search straegy  Results:  12 | (Multiple Sclerosis ) AND (Uveitis ) OR (Retinal Vasculitis) MEDLINE Excluded filter |
