# Supplementary material for: Uveitis characteristics and multiple sclerosis phenotype of patients with multiple sclerosis-associated uveitis: A systematic review and meta-analysis
Source: PLoS One. 2024 Oct 25;19(10):e0307455. doi: 10.1371/journal.pone.0307455 (PMC11508149; doi:10.1371/journal.pone.0307455)
Supplement: S2 File — (DOCX) [file pone.0307455.s003.docx]

**Supporting information 3: Risk of bias**

**S3 Fig 1 Risk of bias traffic-light plot of cohort studies**


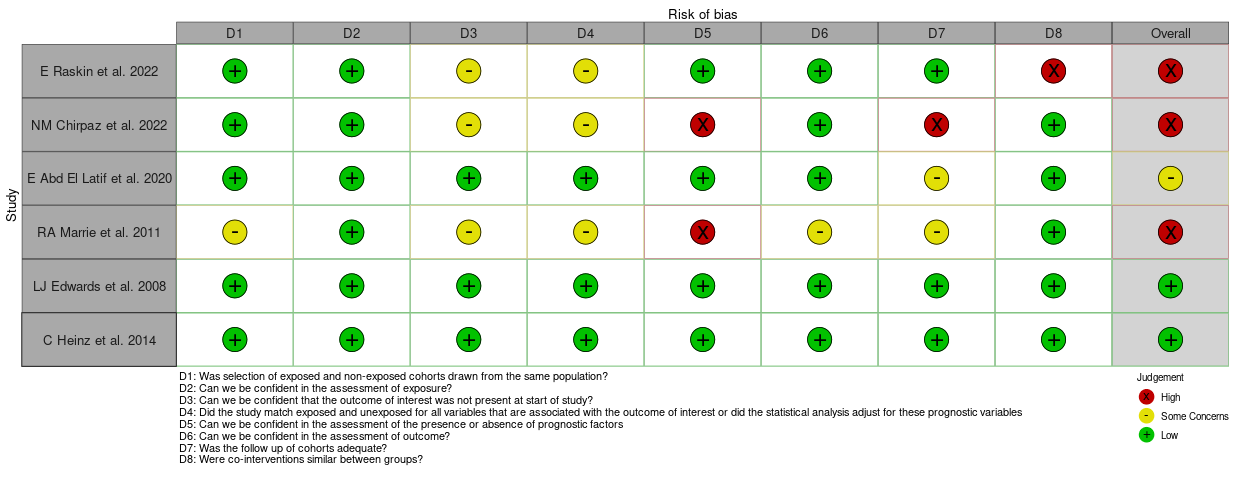


Domain scoring: Definitely yes (low); Probably yes or Probably no (some concern); Definitely no (High).

**S3 Fig 2:** Risk of bias traffic-light plot of case-control studies


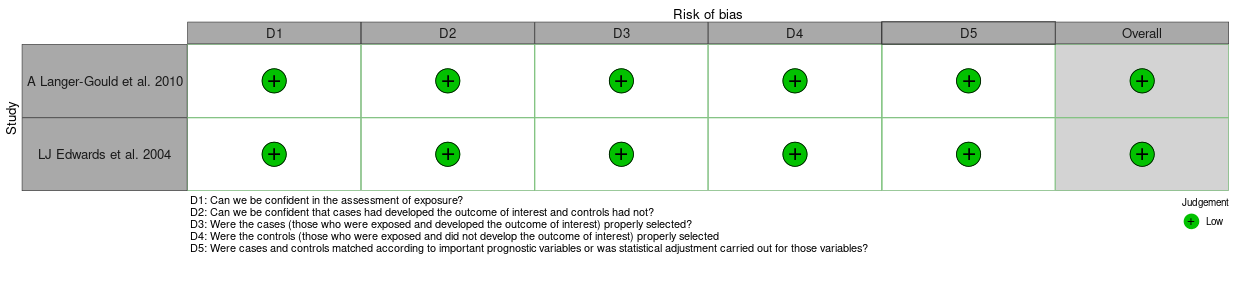


Domain scoring: Definitely yes (low); Probably yes or Probably no (some concern); Definitely no (High).

**S3 Fig 3: Risk of bias traffic-light plot of cross-sectional studies**


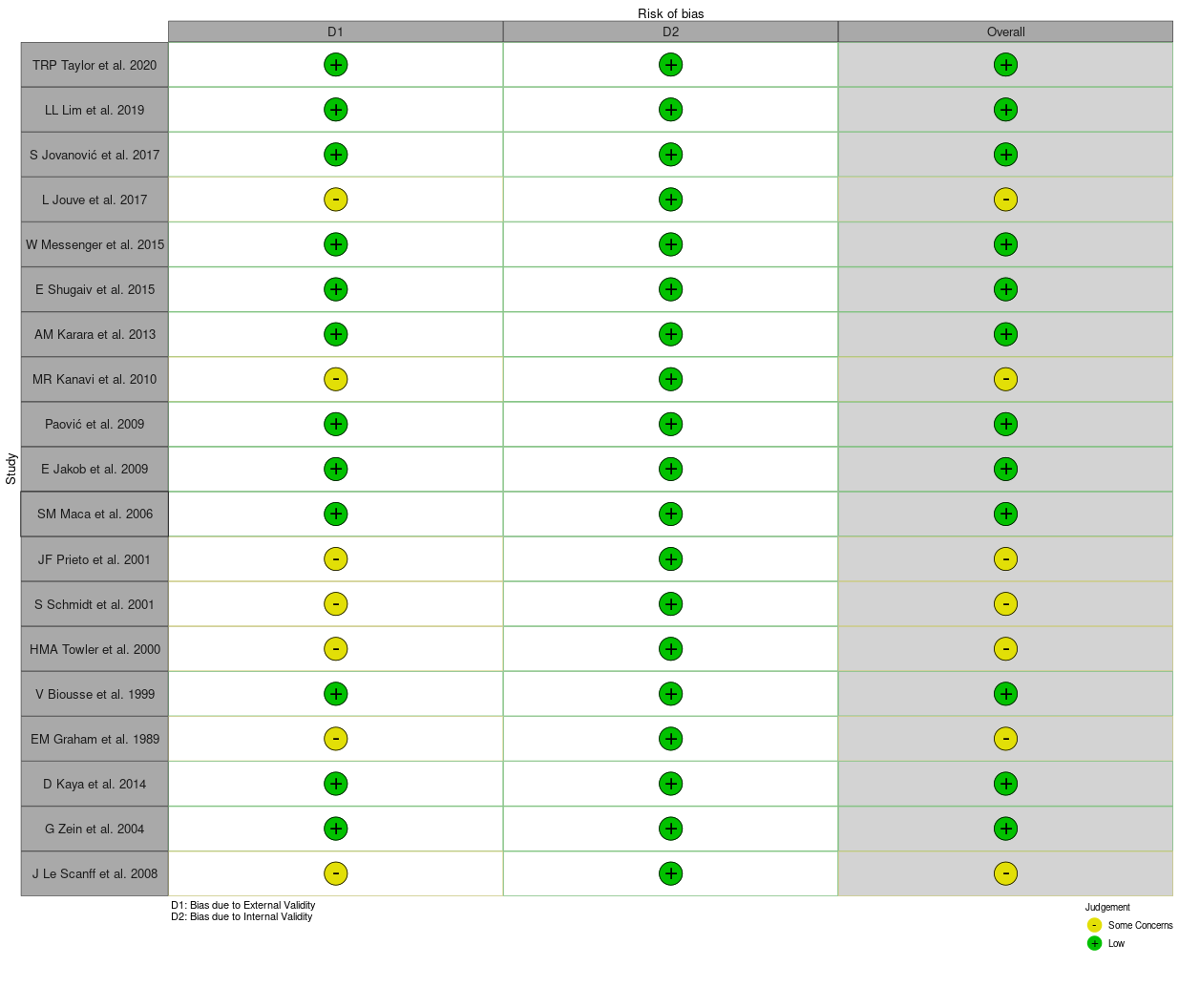


Domain scoring: External Validity - Domain scoring: 0-1 (High); 2(Some Concerns); 3+(Low). Internal Validity - Domain scoring: 0-2 (High); 3(Some Concerns); 4+(Low).

**S3 Fig 4: Risk of bias of case series**


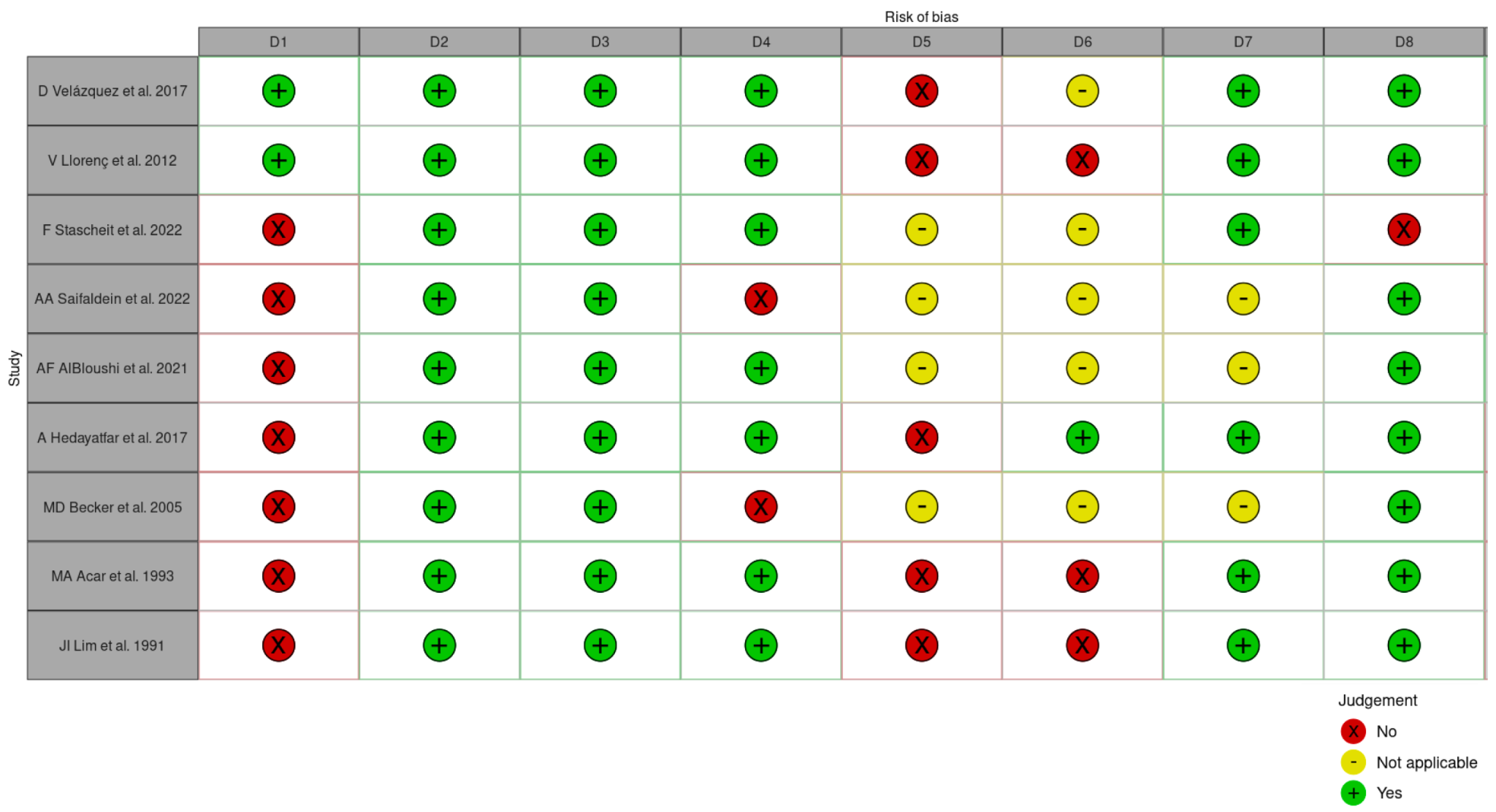


D1: Does the patient(s) represent(s) the whole experience of the investigator (center) or is the selection method unclear to the extent that other patients with similar presentation may not have been reported?

D2: Was the exposure adequately ascertained?

D3: Was the outcome adequately ascertained?

D4: Were other alternative causes that may explain the observation ruled out?

D5: Was there a challenge/rechallenge phenomenon?

D6: Was there a dose–response effect?

D7: Was follow-up long enough for outcomes to occur?

D8: Is the case(s) described with sufficient details to allow other investigators to replicate the research or to allow practitioners make inferences related to their own practice?
