# Supplementary material for: Uveitis characteristics and multiple sclerosis phenotype of patients with multiple sclerosis-associated uveitis: A systematic review and meta-analysis
Source: PLoS One. 2024 Oct 25;19(10):e0307455. doi: 10.1371/journal.pone.0307455 (PMC11508149; doi:10.1371/journal.pone.0307455)

**Supporting Information 4: Sensitivity analysis**

**1 Pool prevalence Metanalysis**

**Demographic and ocular clinical characteristics**

**S4 Fig 1: Overall female prevalence**


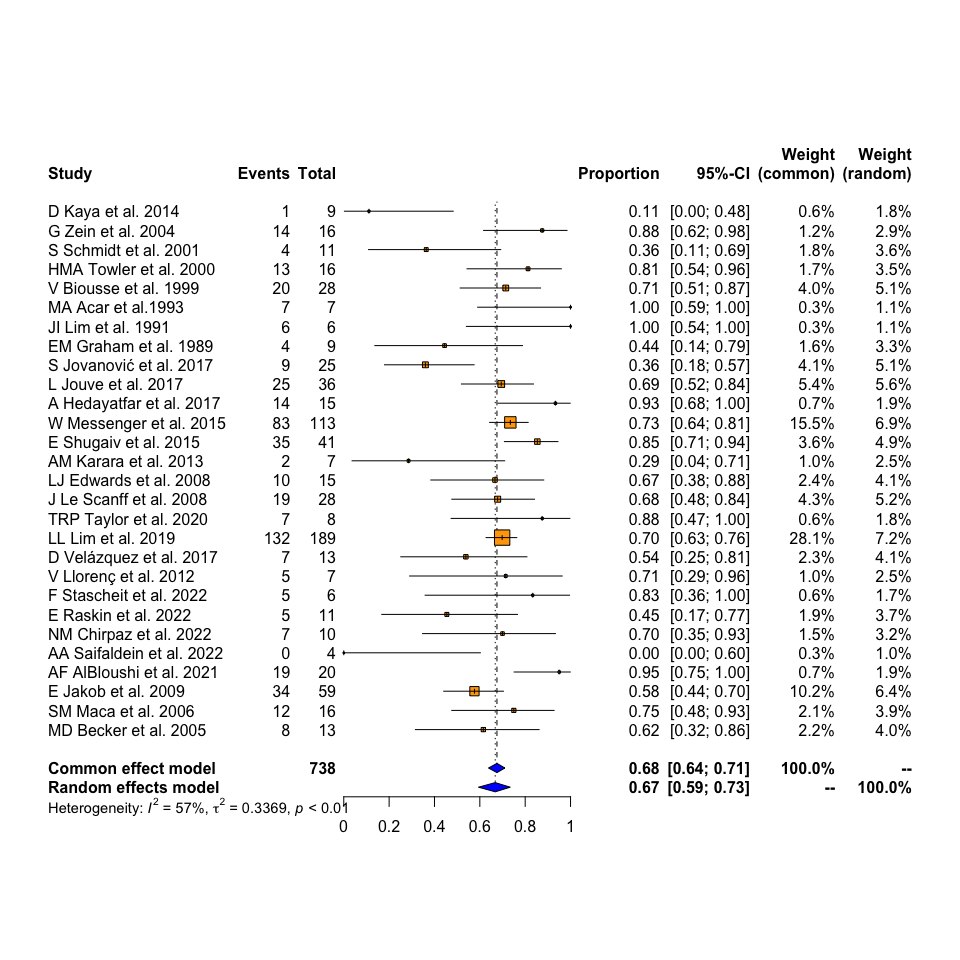


**S4 Fig 2: Overall male prevalence**
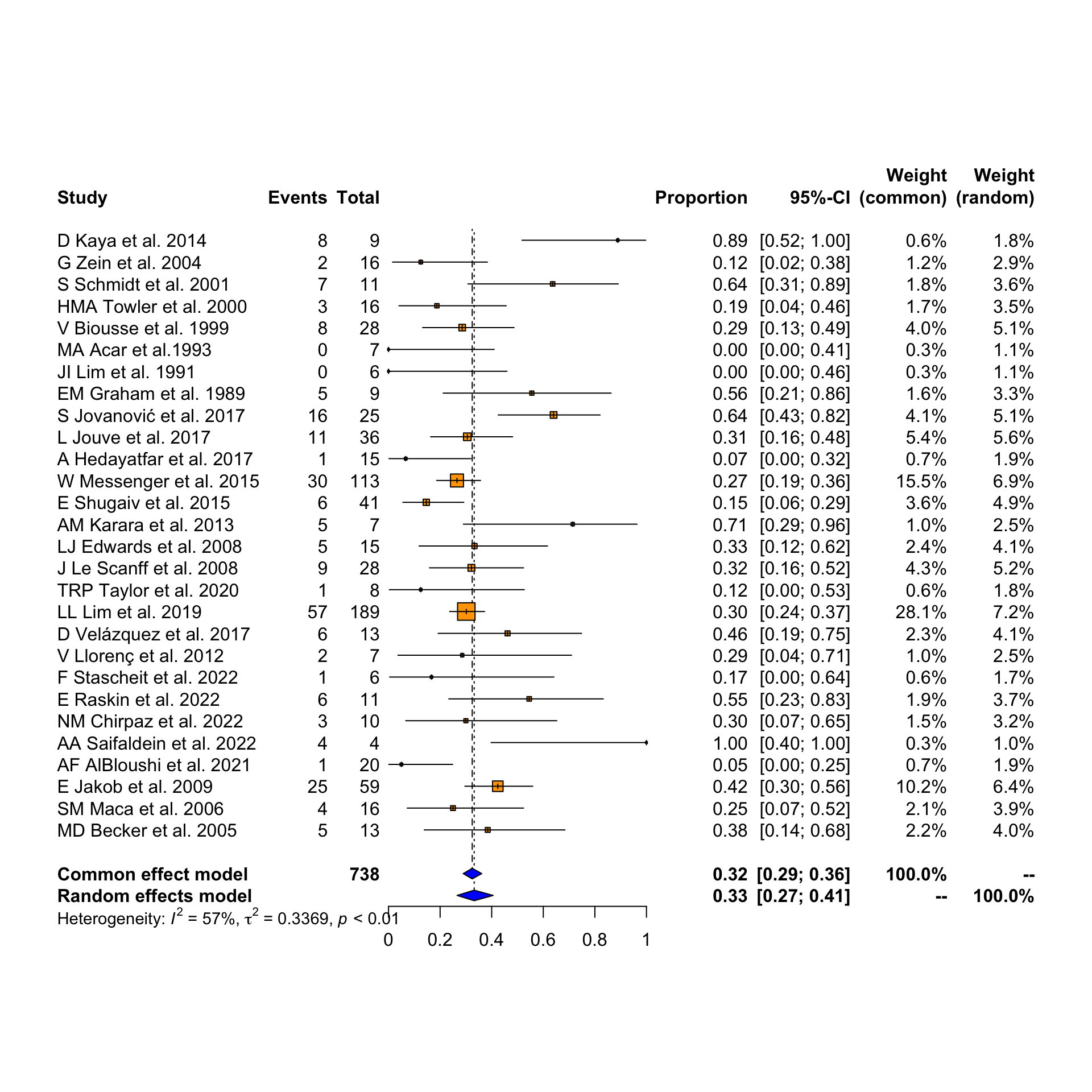


**S4 Fig 3: Prevalence of patients who presented uveitis before neurological signs.**


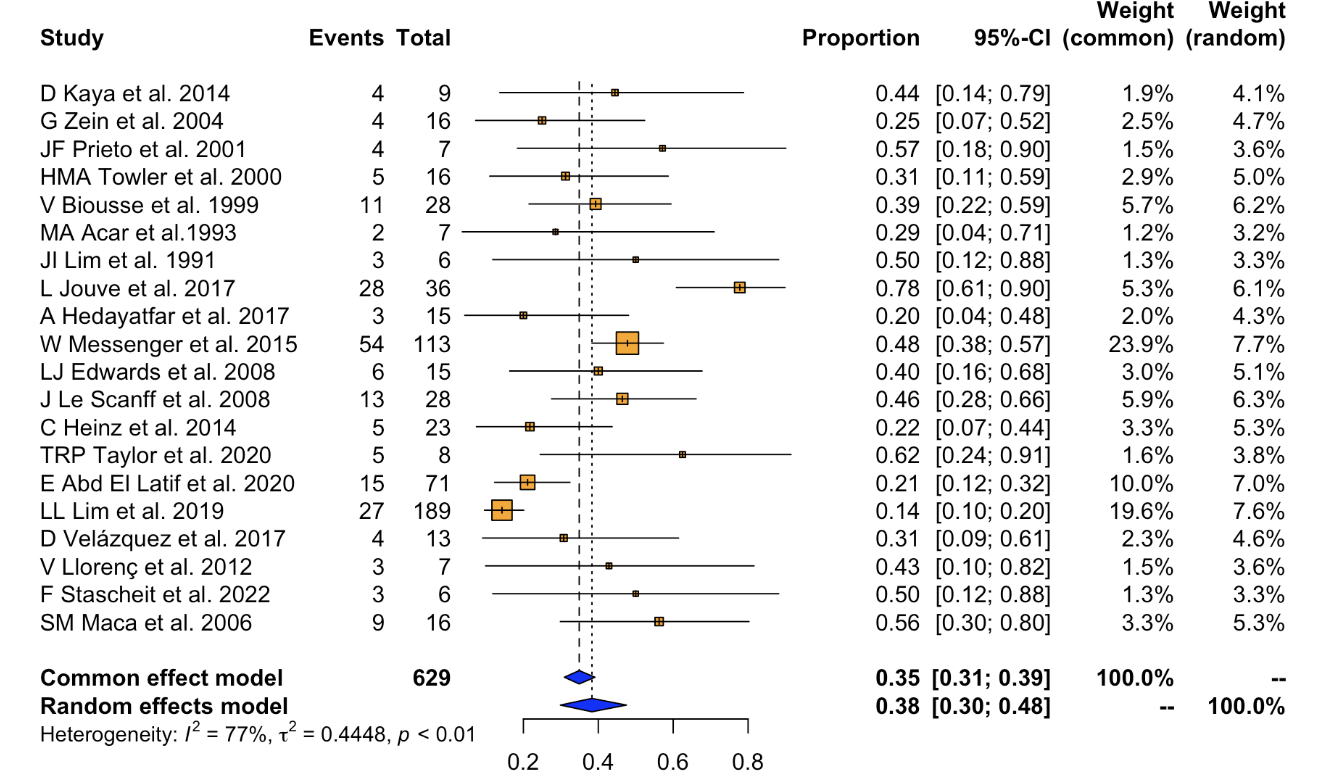


**S4 Fig 4: Prevalence of patients who presented uveitis between 1-10 years.**


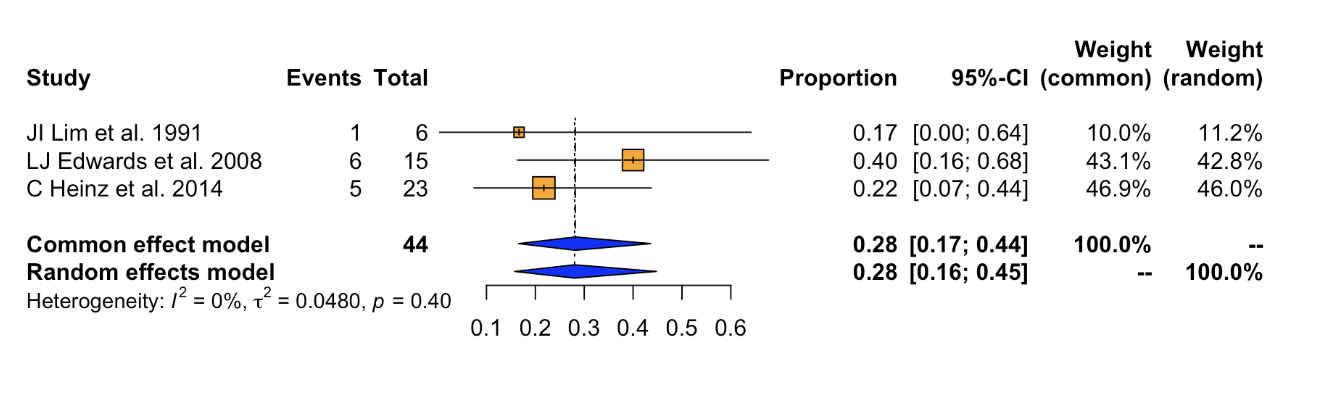


**S4 Fig 5: Prevalence of patients who presented uveitis between 11-20 years.**


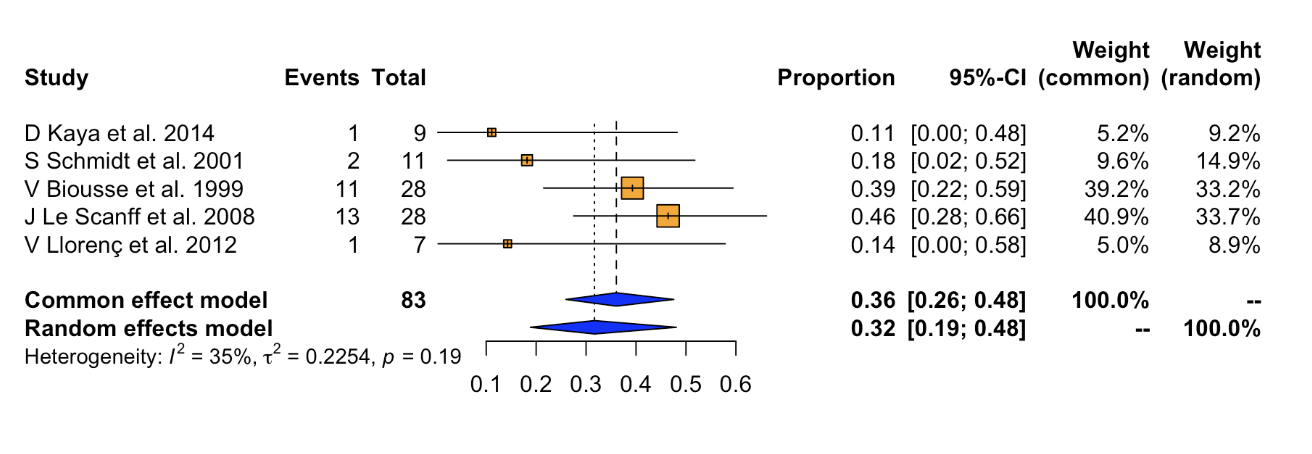


**S4 Fig 6: Prevalence of patients who presented uveitis between 21-30 years.**


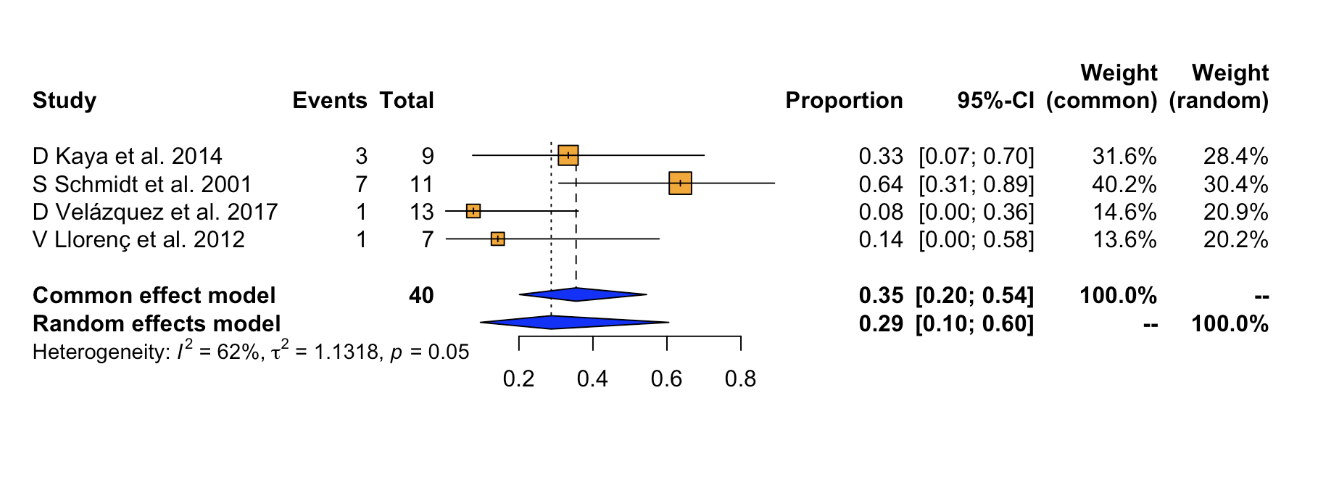


**S4 Fig 7: Prevalence of patients who presented uveitis between 31-40 years.**


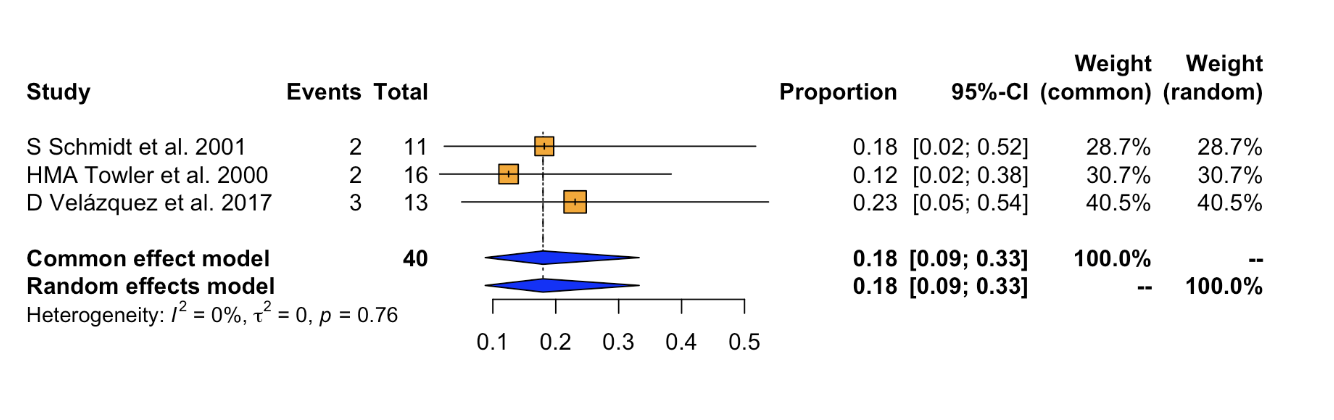


**S4 Fig 8: Prevalence of patients who presented uveitis between 41-50 years.**


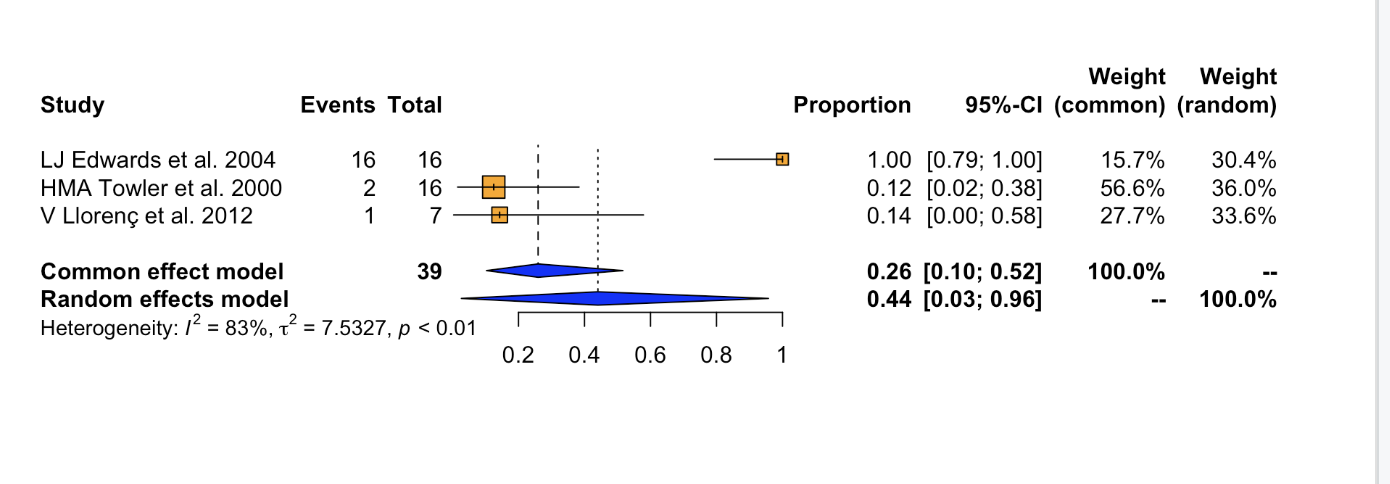


**S4 Fig 9: Prevalence of patients who presented uveitis between 51-60 years.**


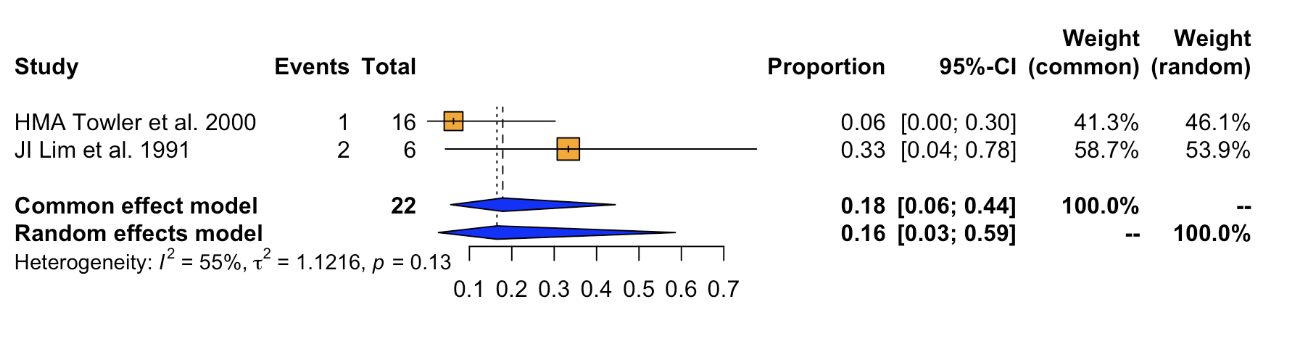


**S4 Fig 10: Prevalence of patients who presented unilateral uveitis.**


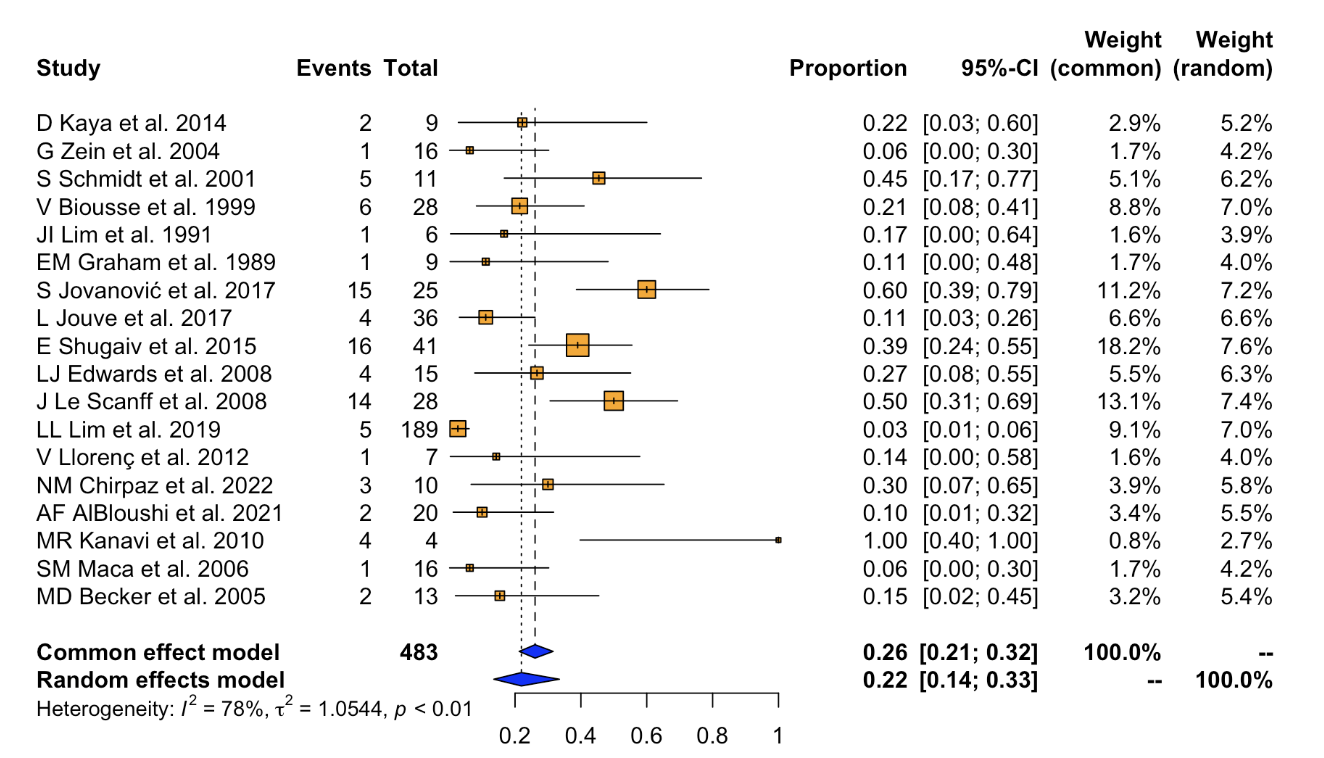


**S4 Fig 11: Prevalence of patients who presented bilateral uveitis.**


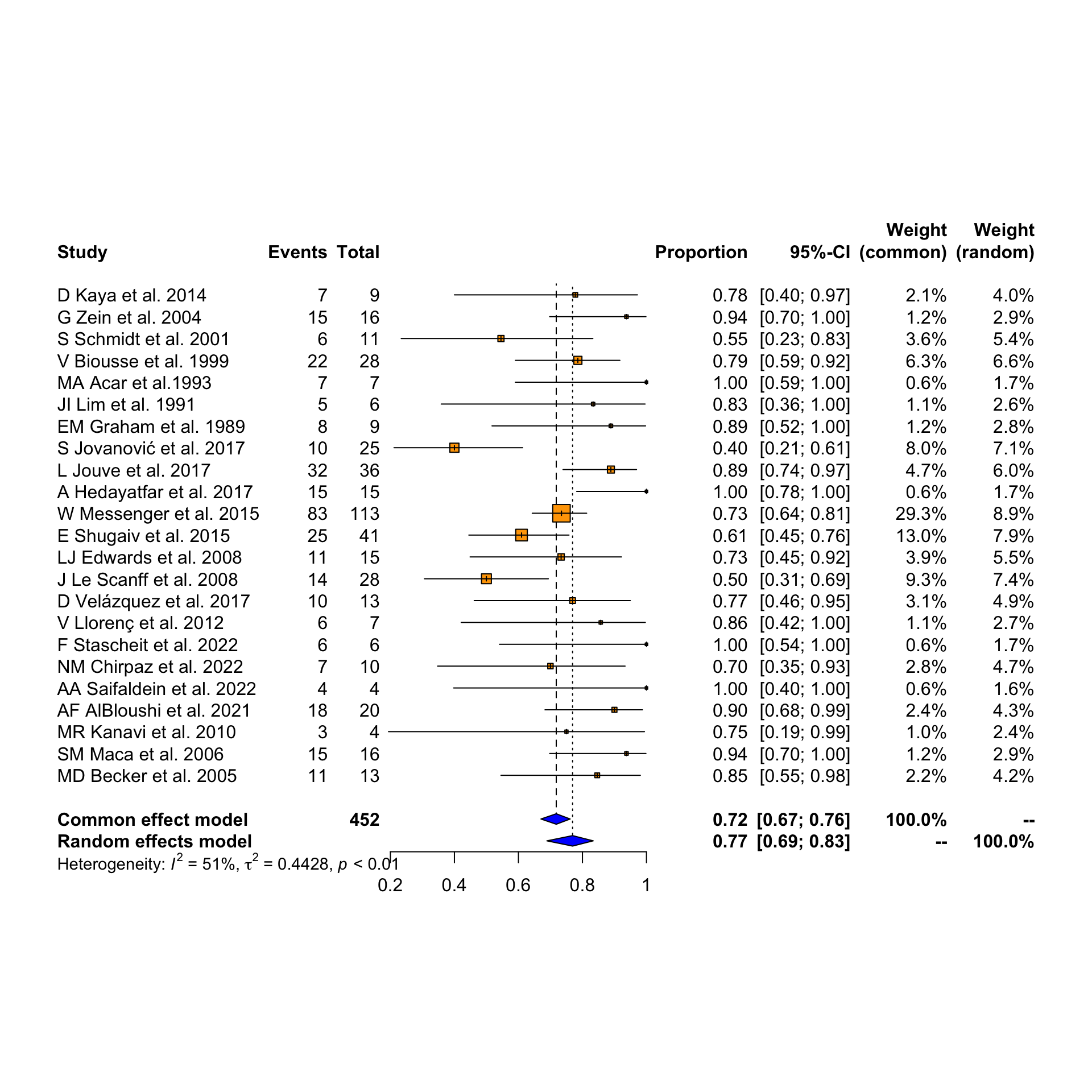


**S4 Fig 12: Prevalence of patients initially presenting with unilateral uveitis progressing to bilateral uveitis**


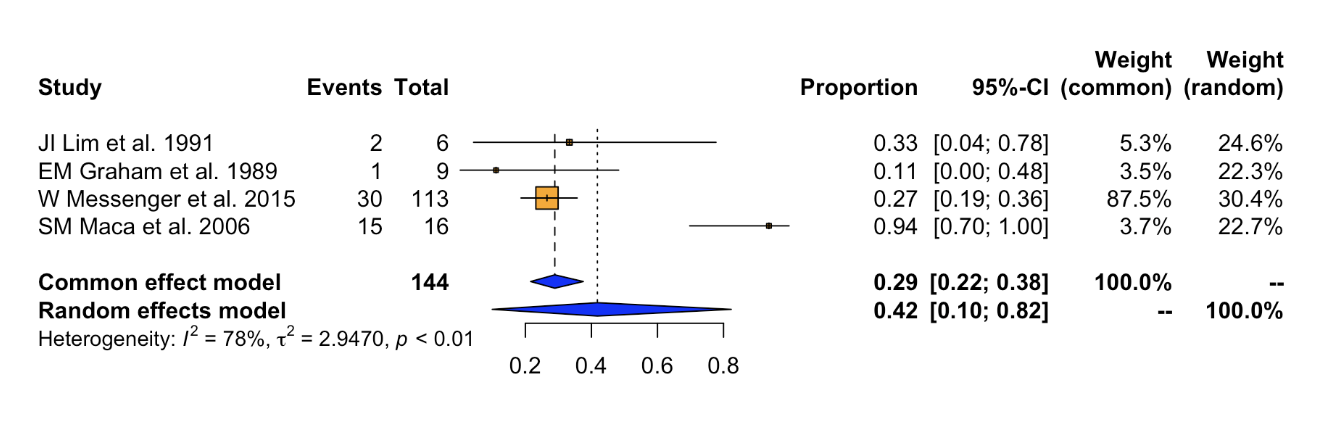


**S4 Fig 13: Prevalence of patients with anterior uveitis**


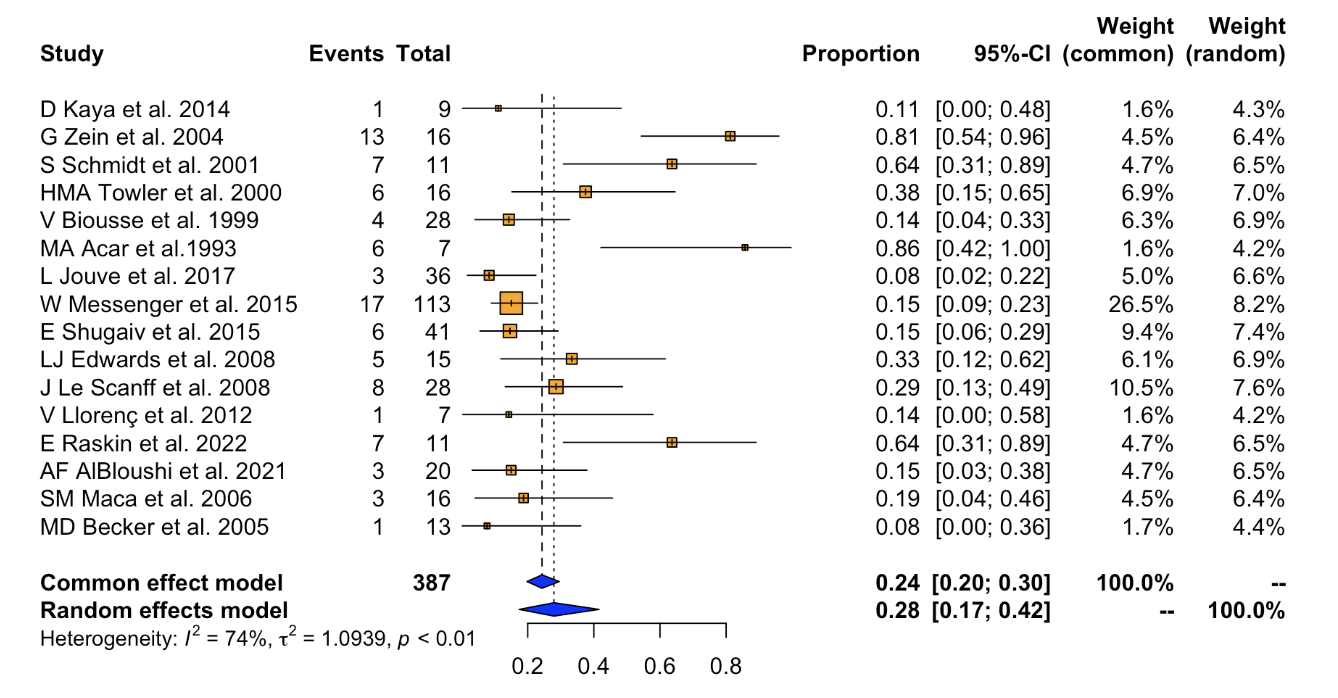


**S4 Fig 14: Prevalence of patients with anterior-intermediate uveitis**


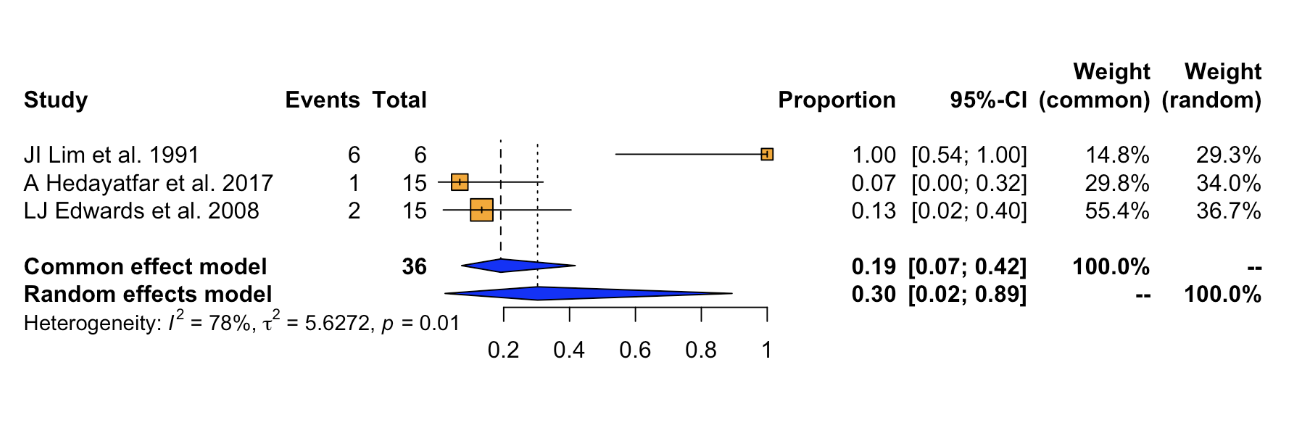


**S4 Fig 15: Prevalence of patients with intermediate uveitis**


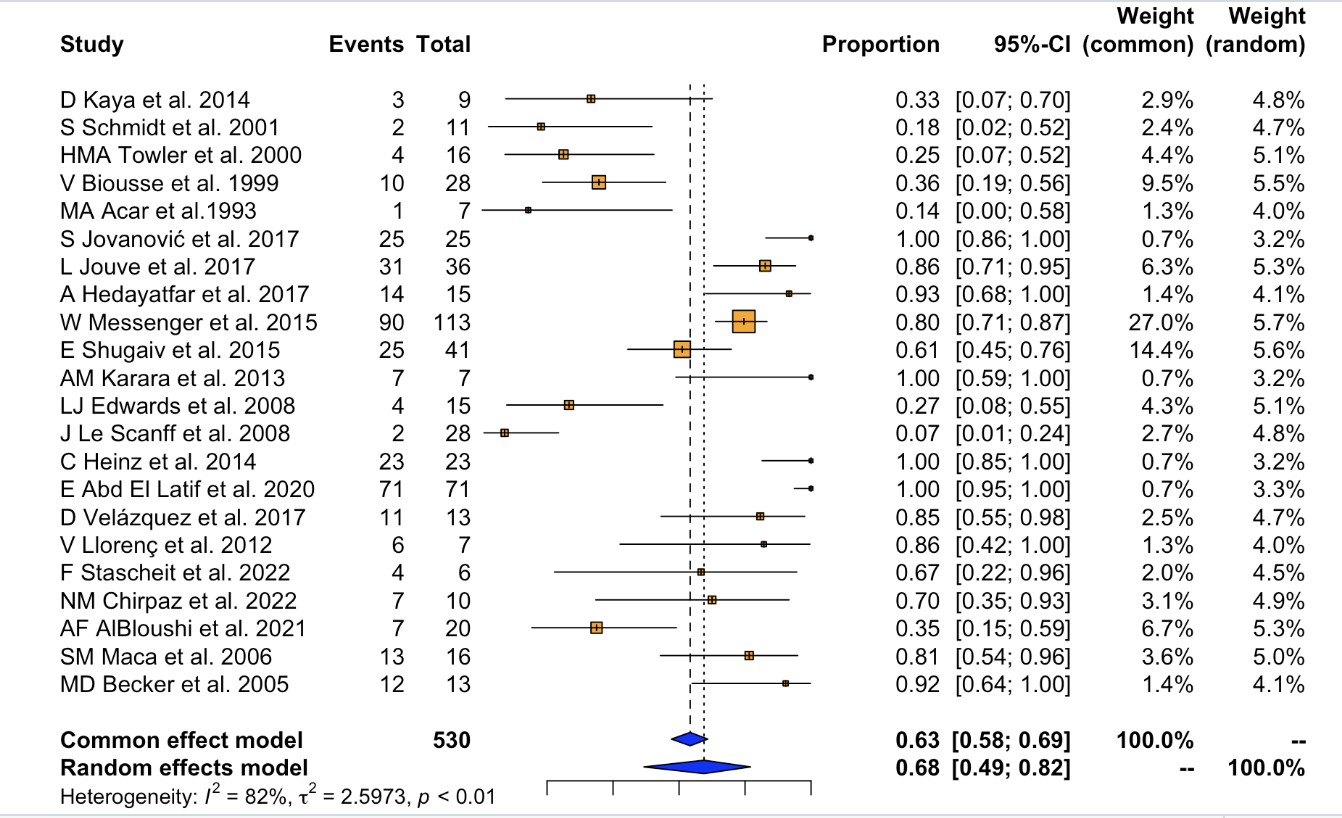


**S4 Fig 16: Prevalence of patients with posterior uveitis**


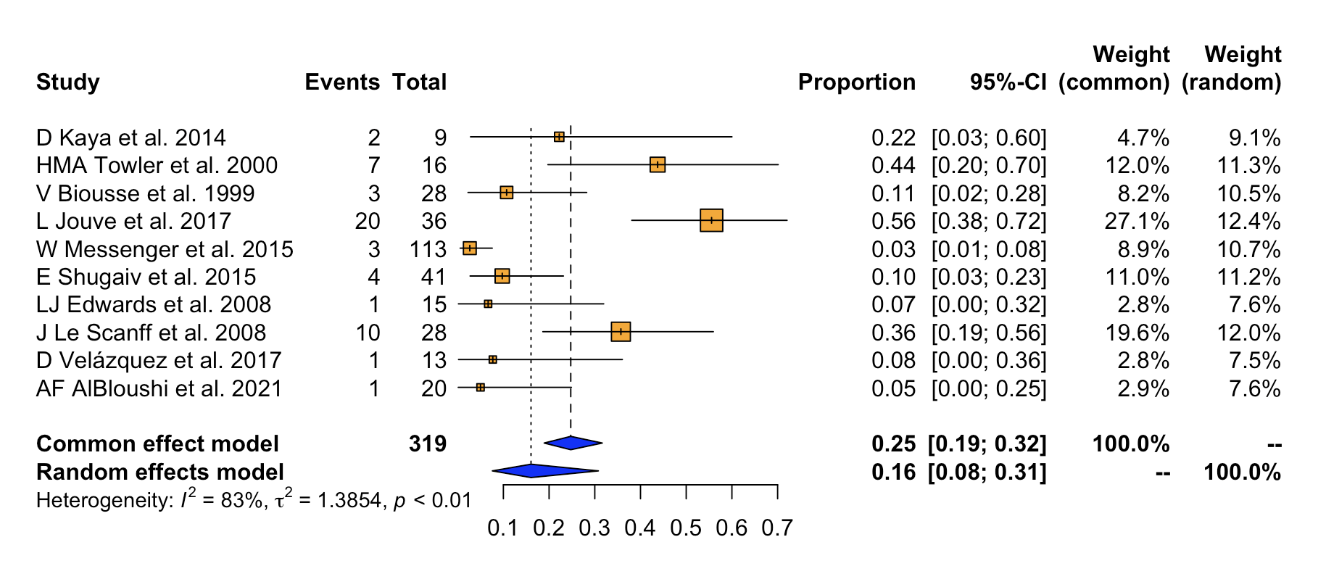


**S4 Fig 17: Prevalence of patients with panuveitis**


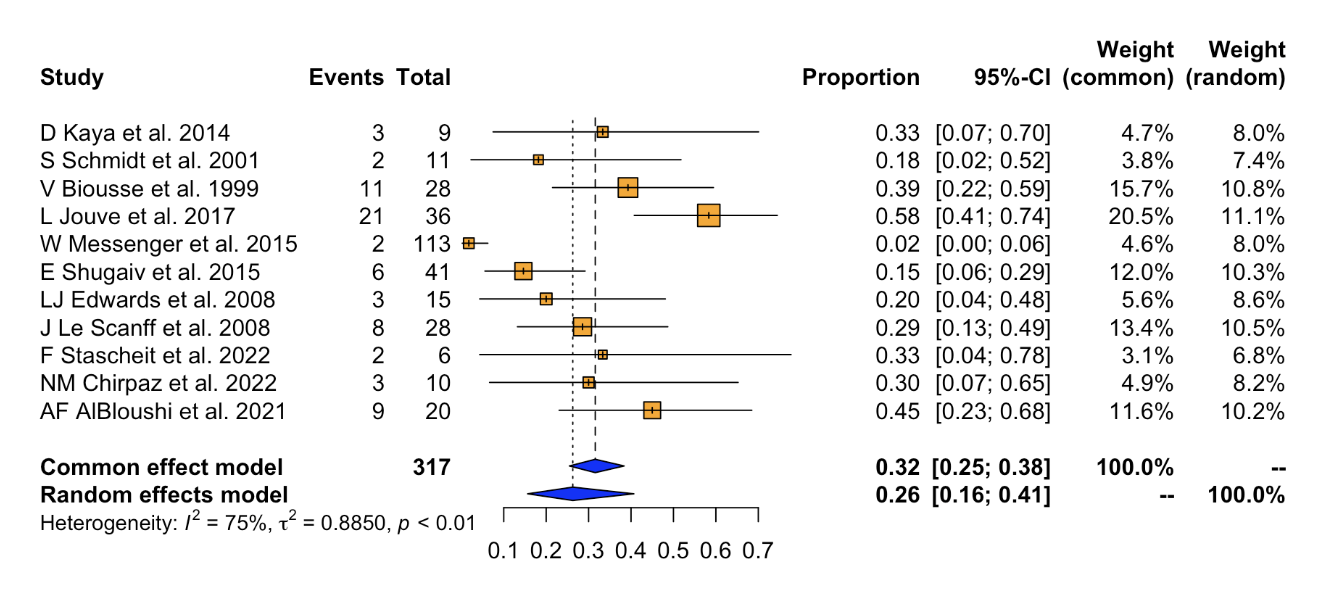


**S4 Fig 18: Prevalence of patients with snowbanks**


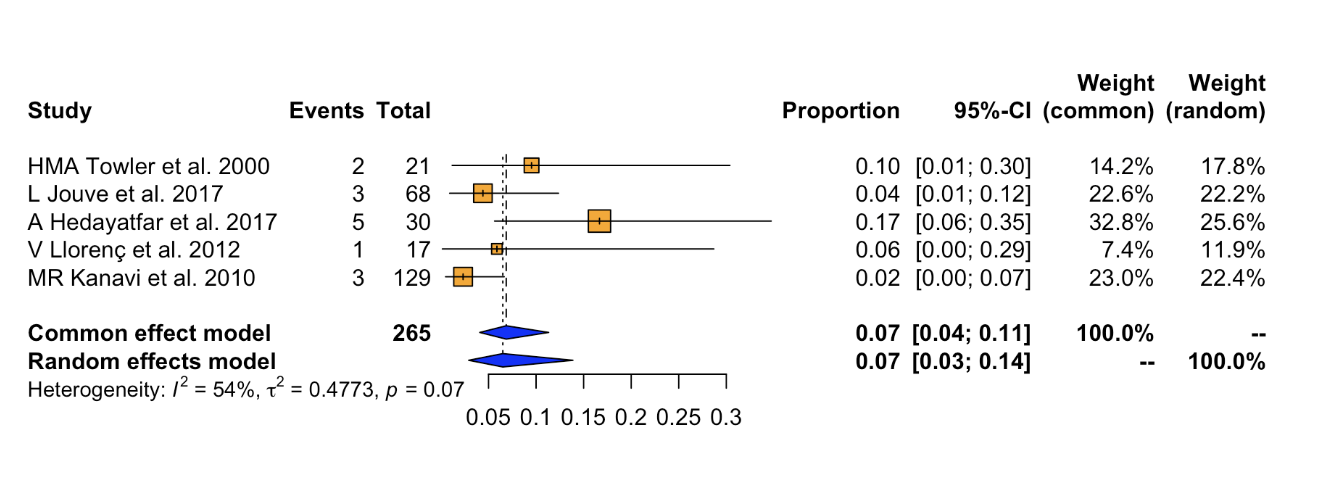


**S4 Fig 19: Prevalence of patients with snowballs**


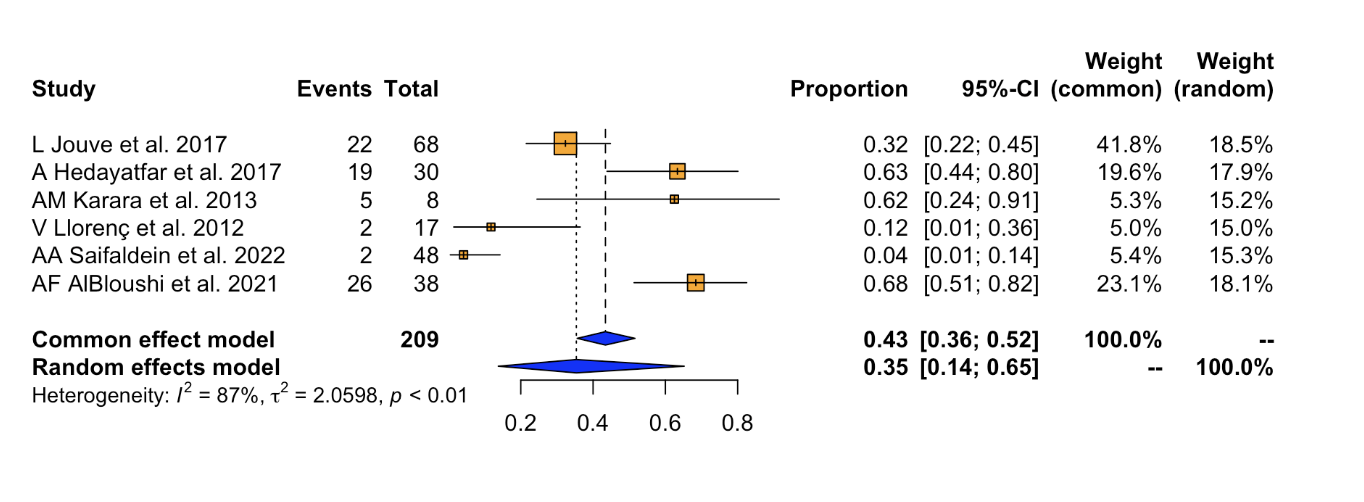


**S4 Fig 20: Prevalence of patients with granulomatous uveitis**


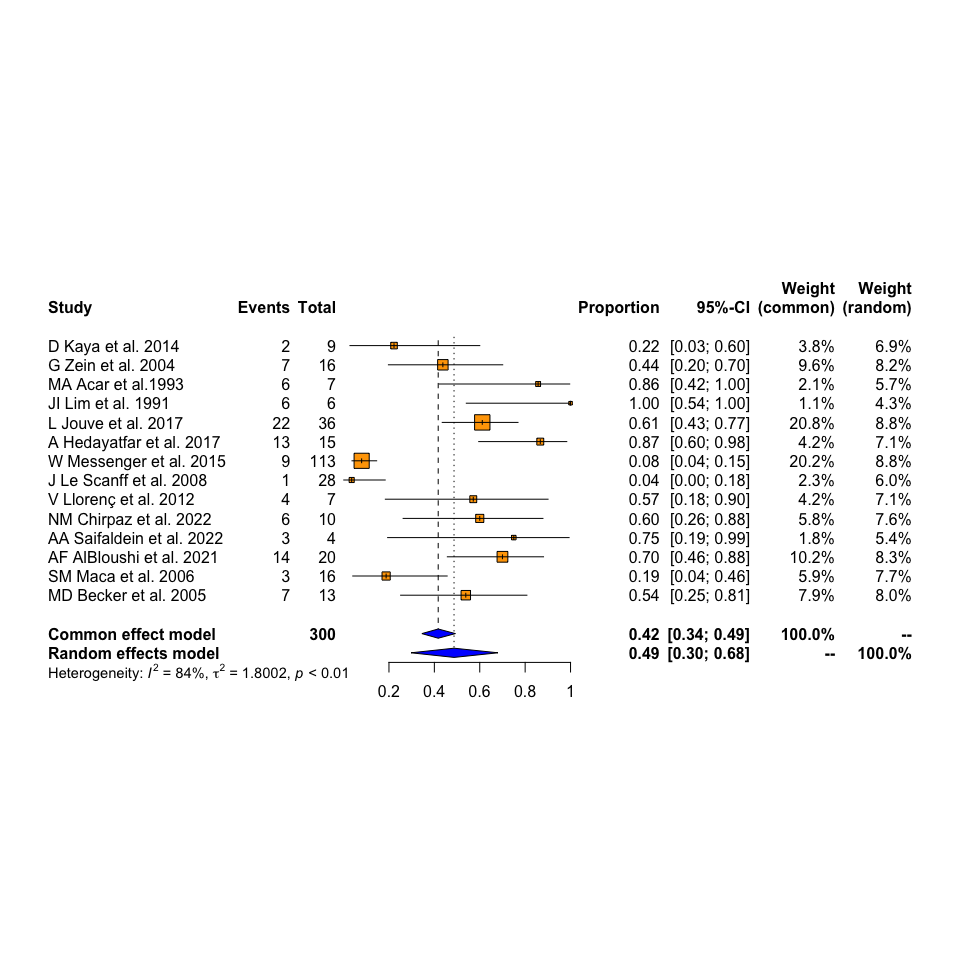


**S4 Fig 21: Prevalence of patients with non-granulomatous uveitis**


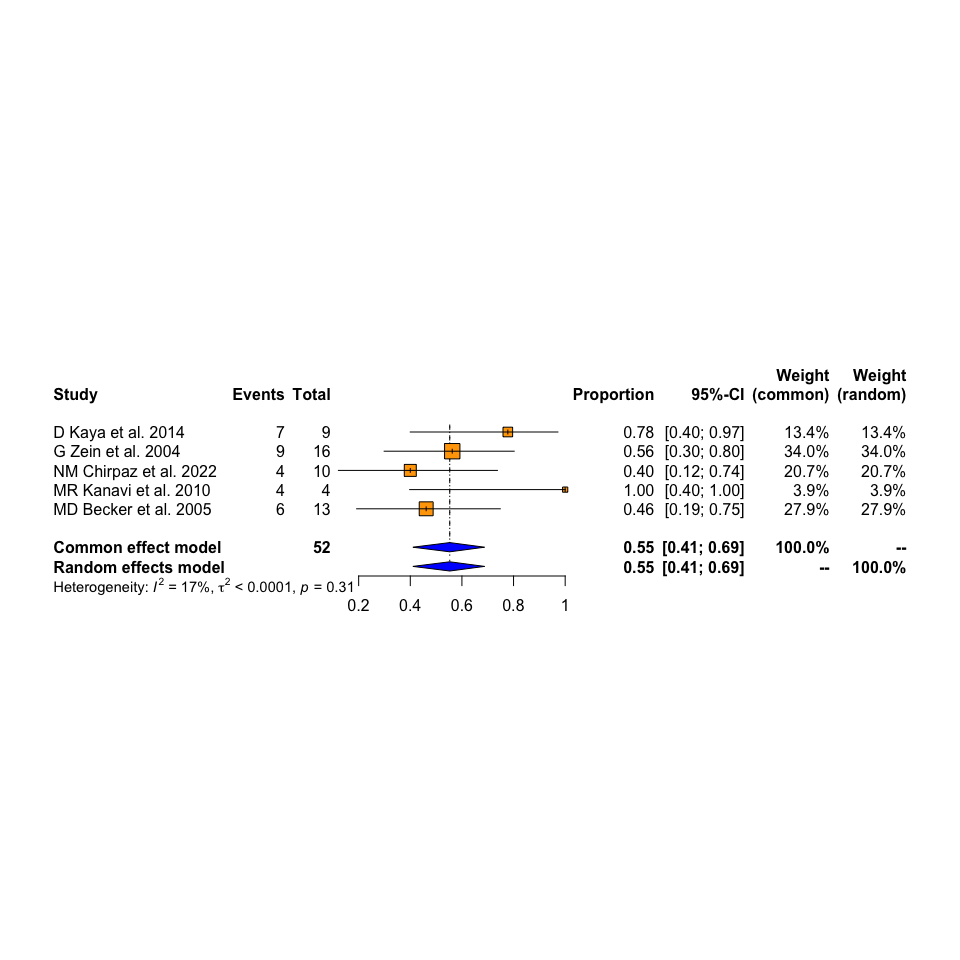


**S4 Fig 22: Prevalence of patients with acute uveitis**


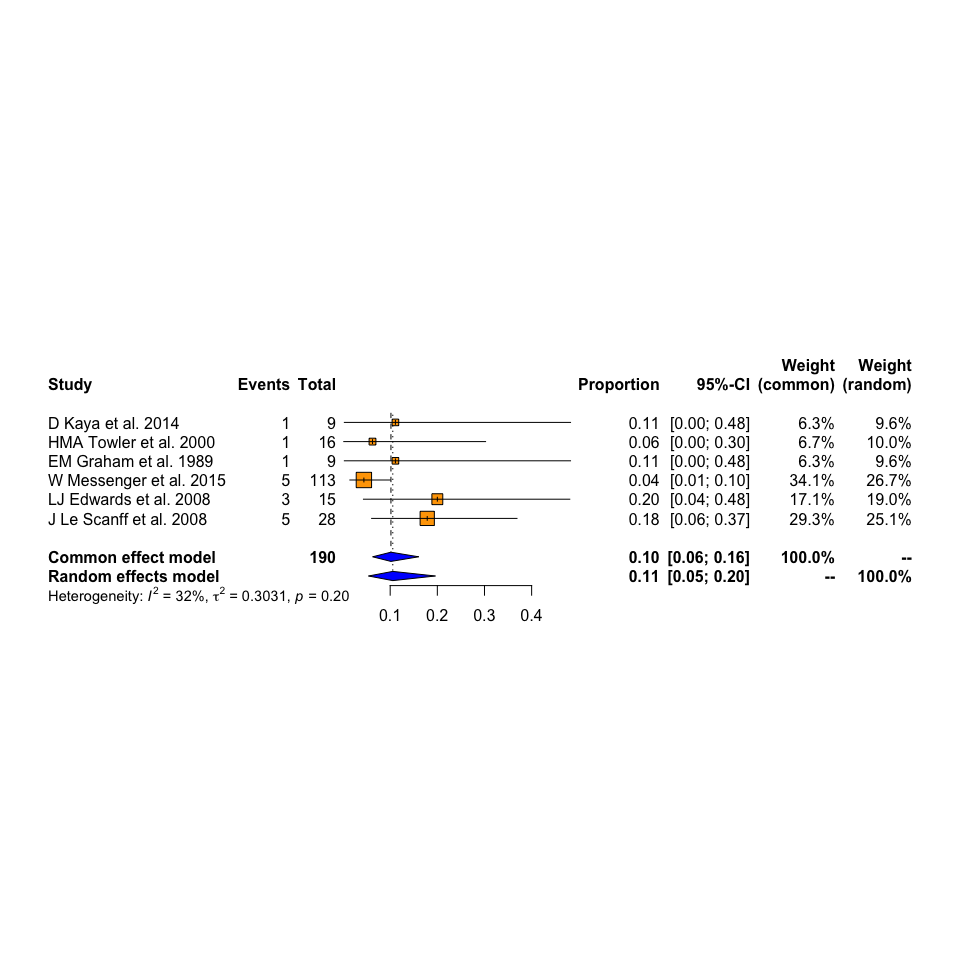


**S4 Fig 23: Prevalence of patients with recurrent uveitis**


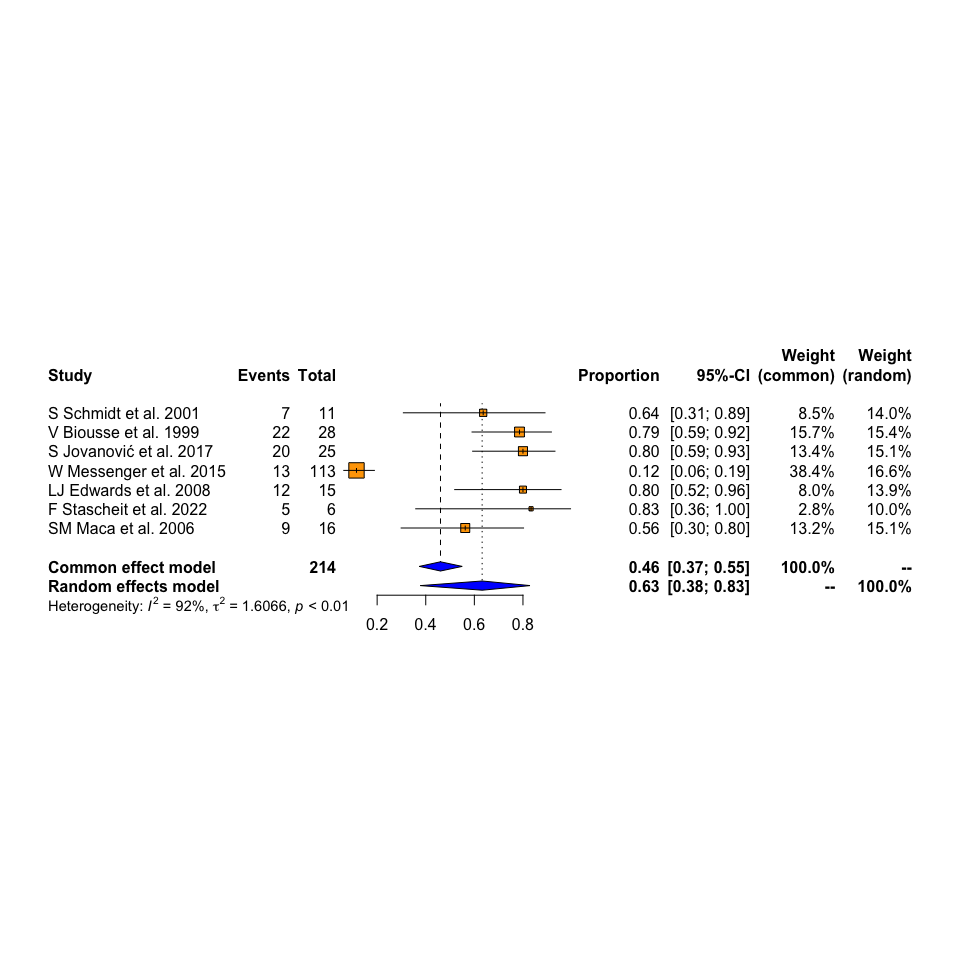


**S4 Fig 24: Prevalence of patients with chronic uveitis**

​​
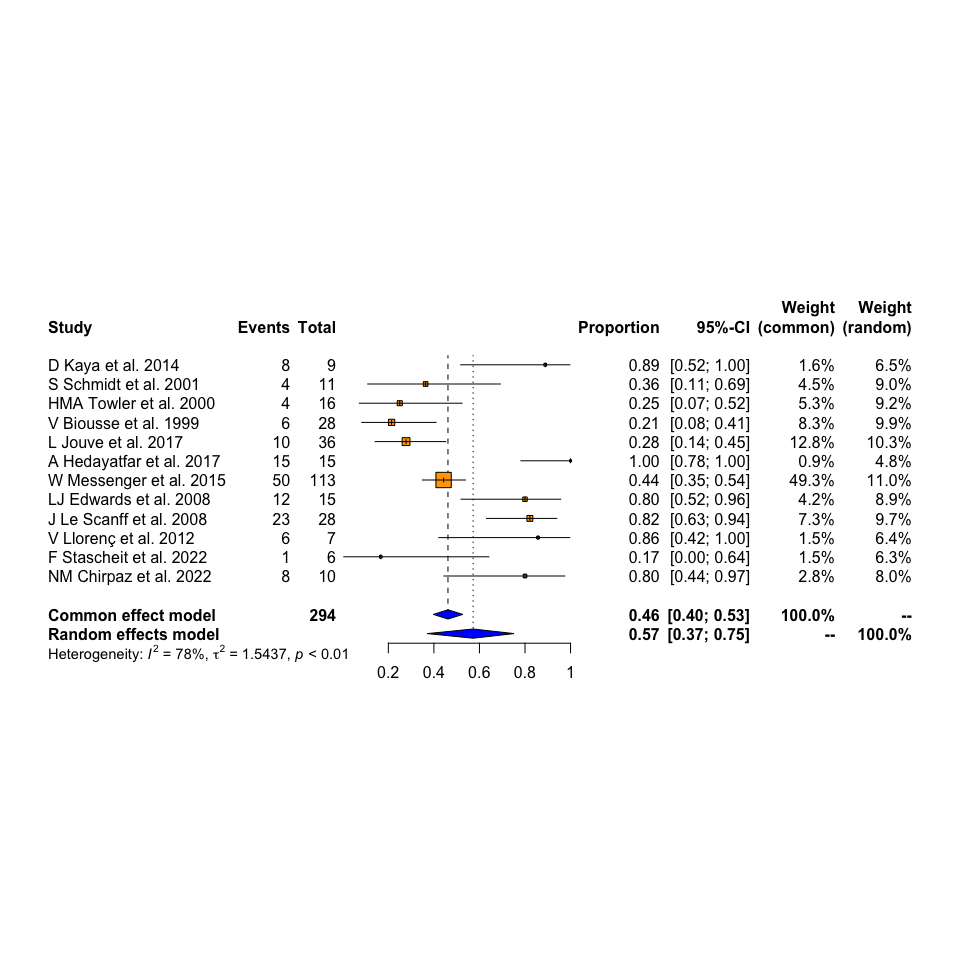


**S4 Fig 25: Prevalence of patients with decreased vision**

**
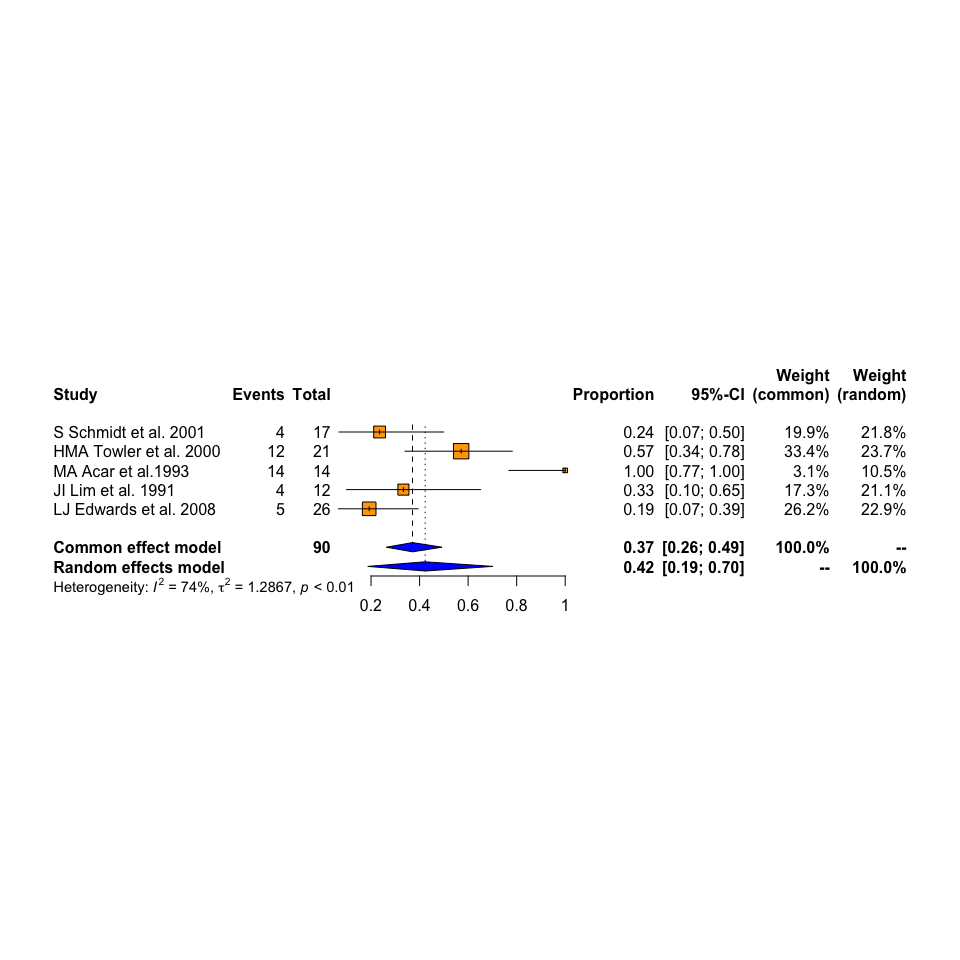
**

**S4 Fig 26: Prevalence of patients with glaucoma**


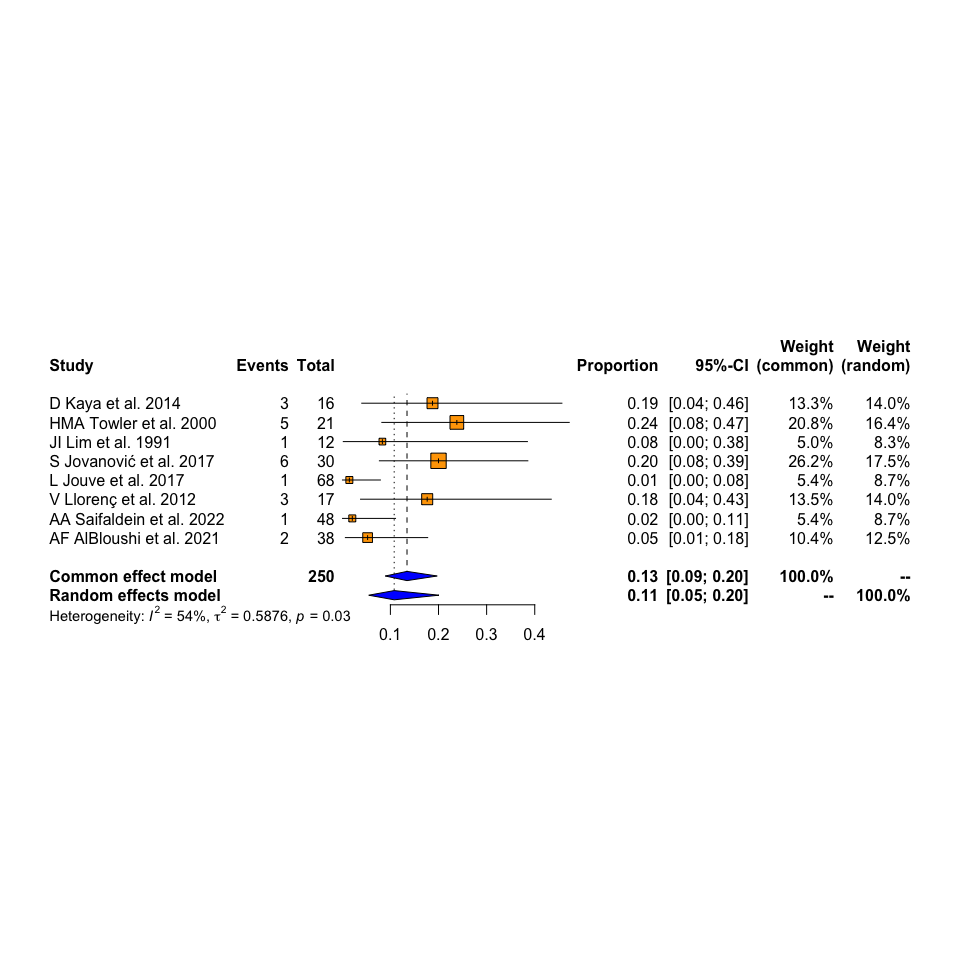


**S4 Fig 27: Prevalence of patients with periphlebitis**


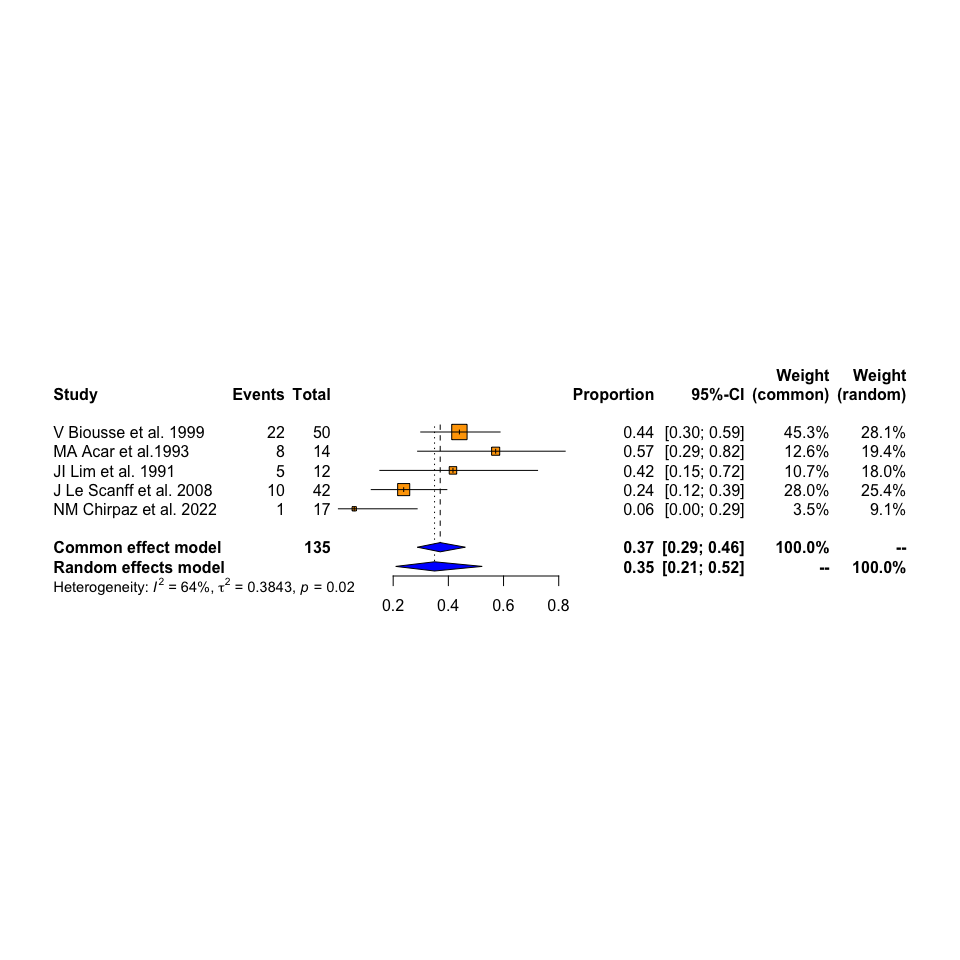


**S4 Fig 28: Prevalence of patients with vascular sheating**


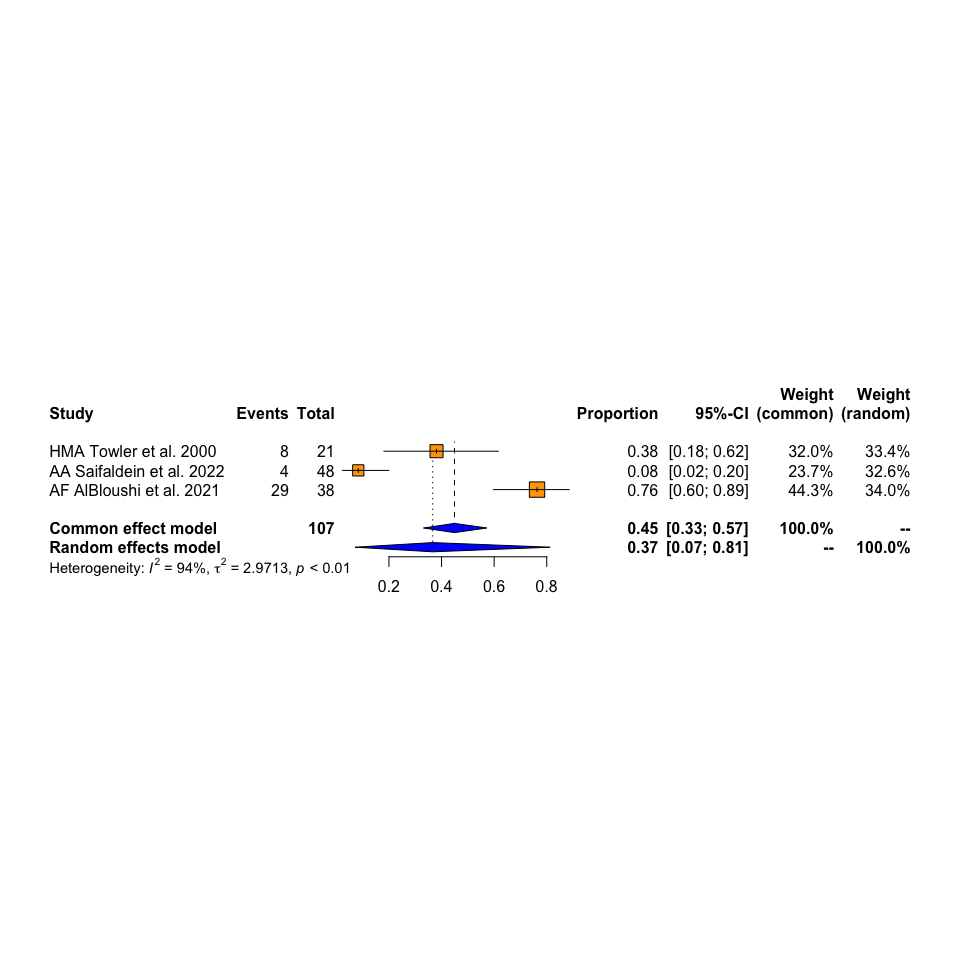


**S4 Fig 29: Prevalence of patients with macular changes**


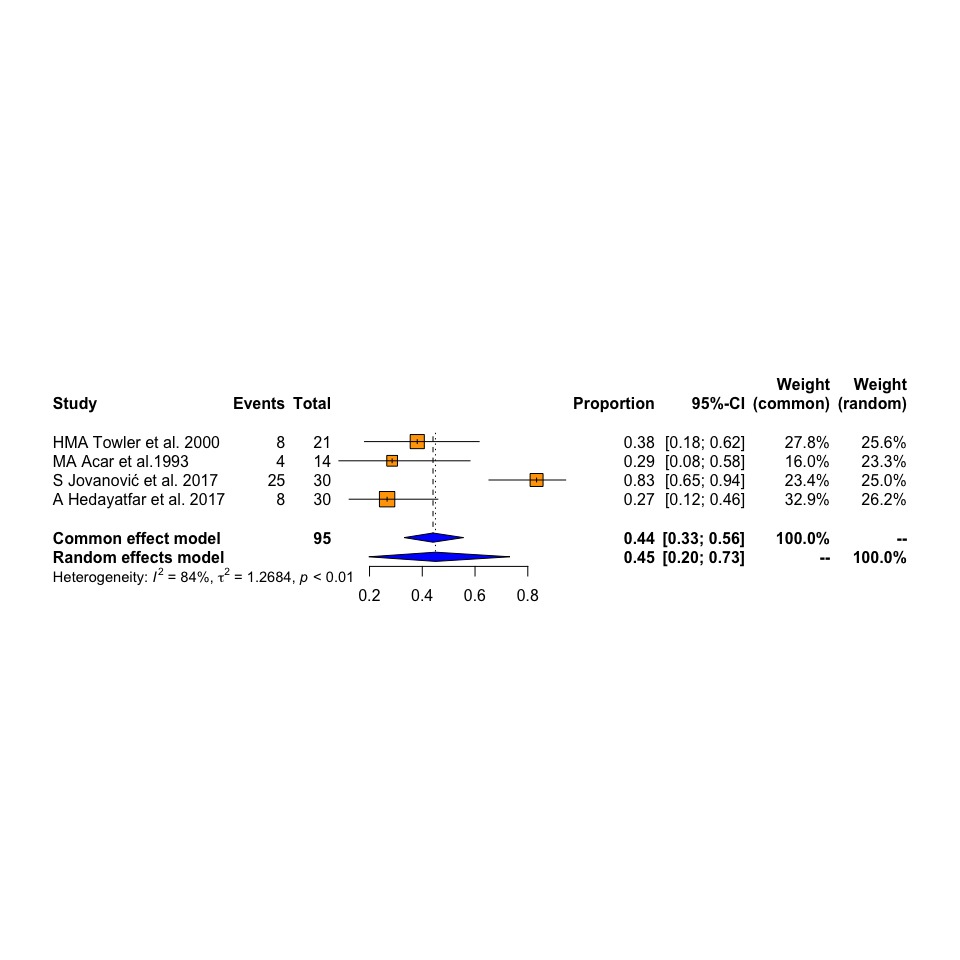


**S4 Fig 30: Prevalence of patients with macular edema**


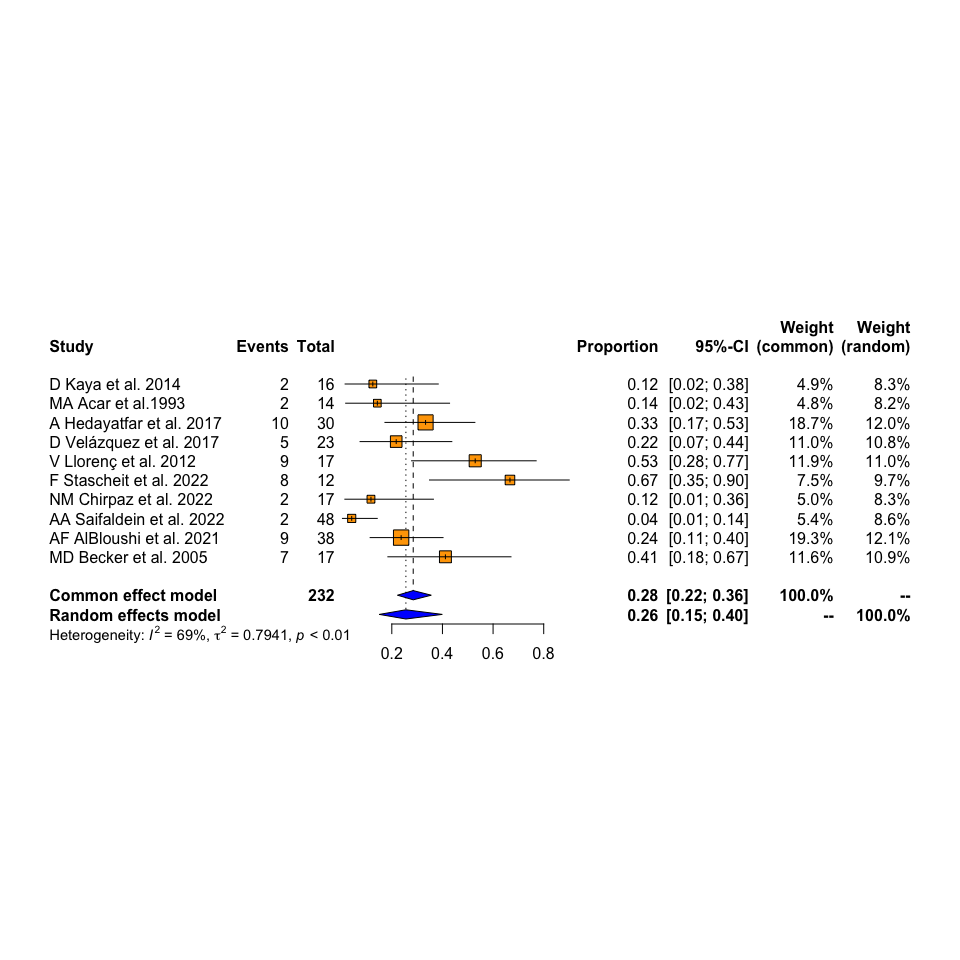


**S4 Fig 31: Prevalence of patients with epiretinal membrane**


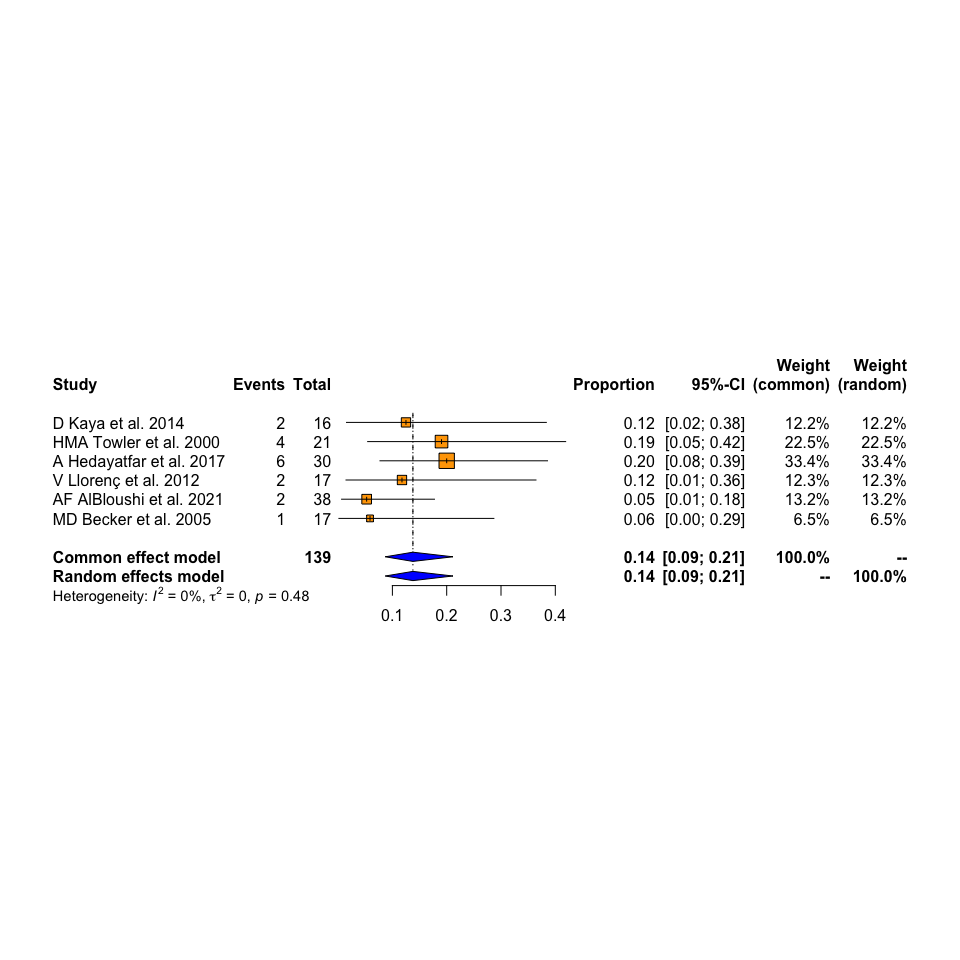


**S4 Fig 32: Prevalence of patients with cataract**


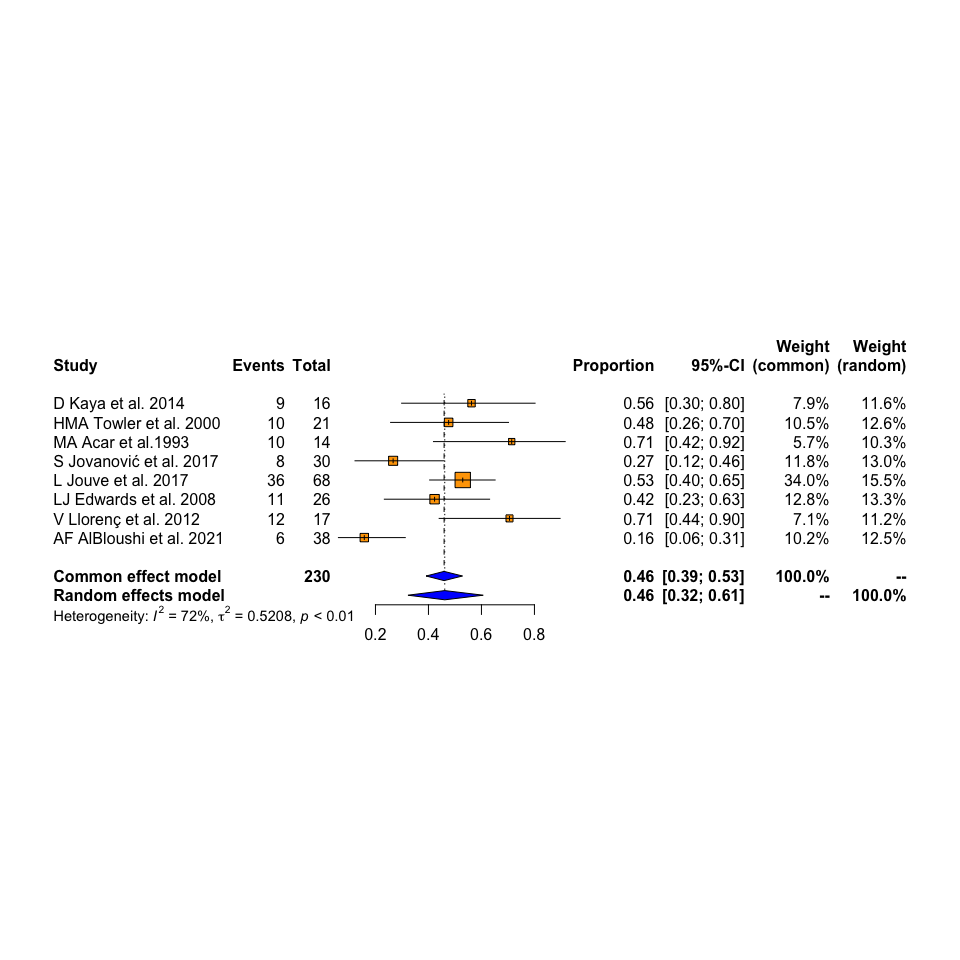


**S4 Fig 33: Prevalence of patients with retinal detatchment**


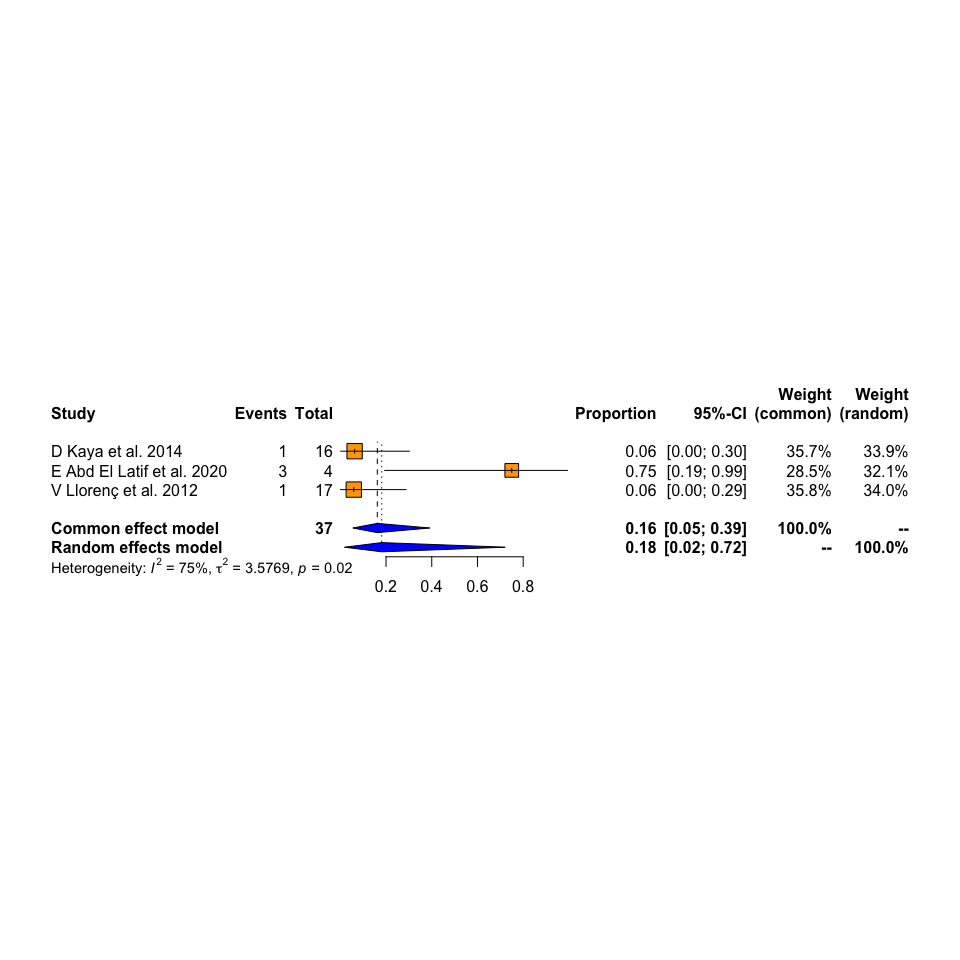


**S4 Fig 34: Prevalence of patients with vitreous hemorrhage**


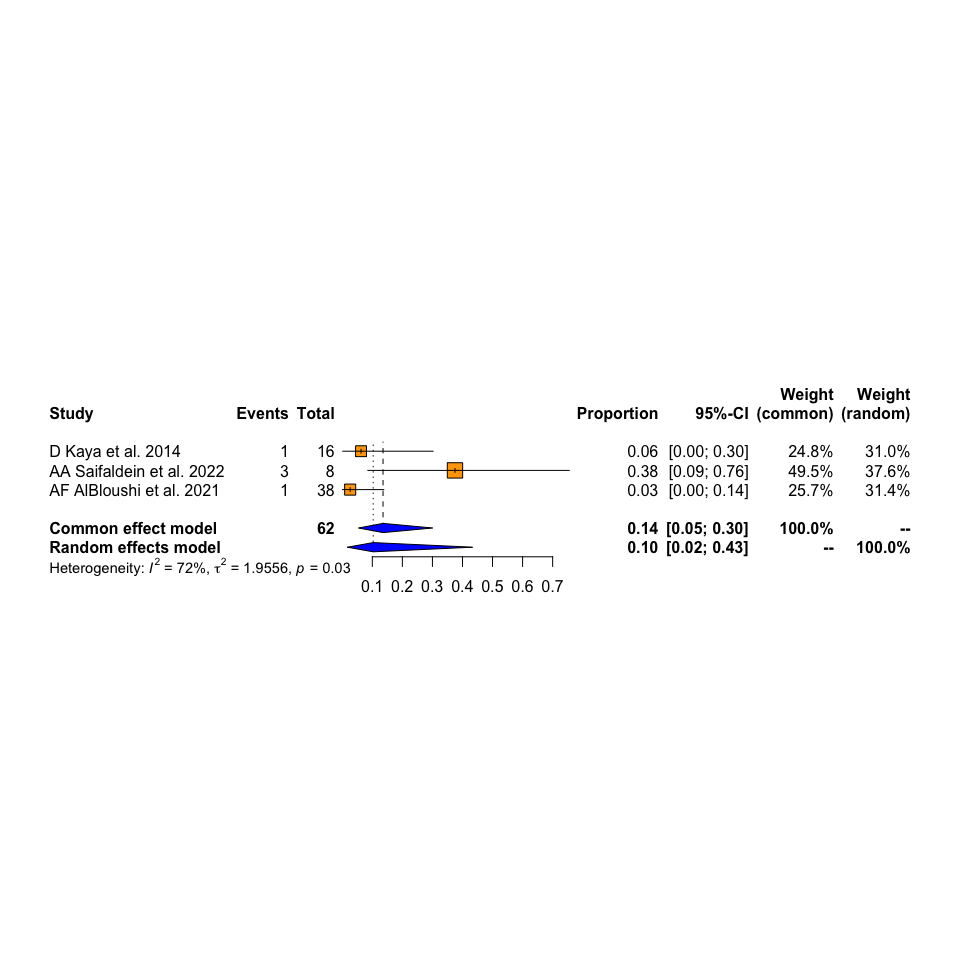


**S4 Fig 35: Prevalence of patients with keratic precipitates**


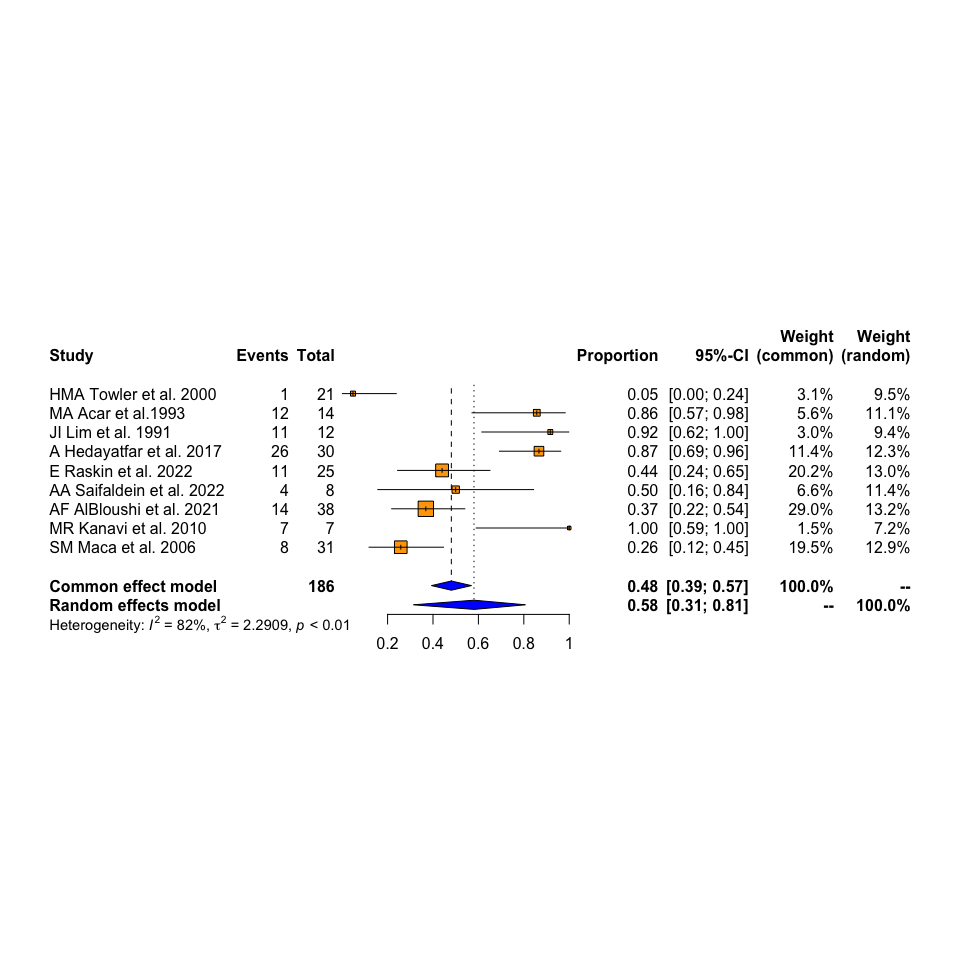


**S4 Fig 36: Prevalence of patients with iris nodules**


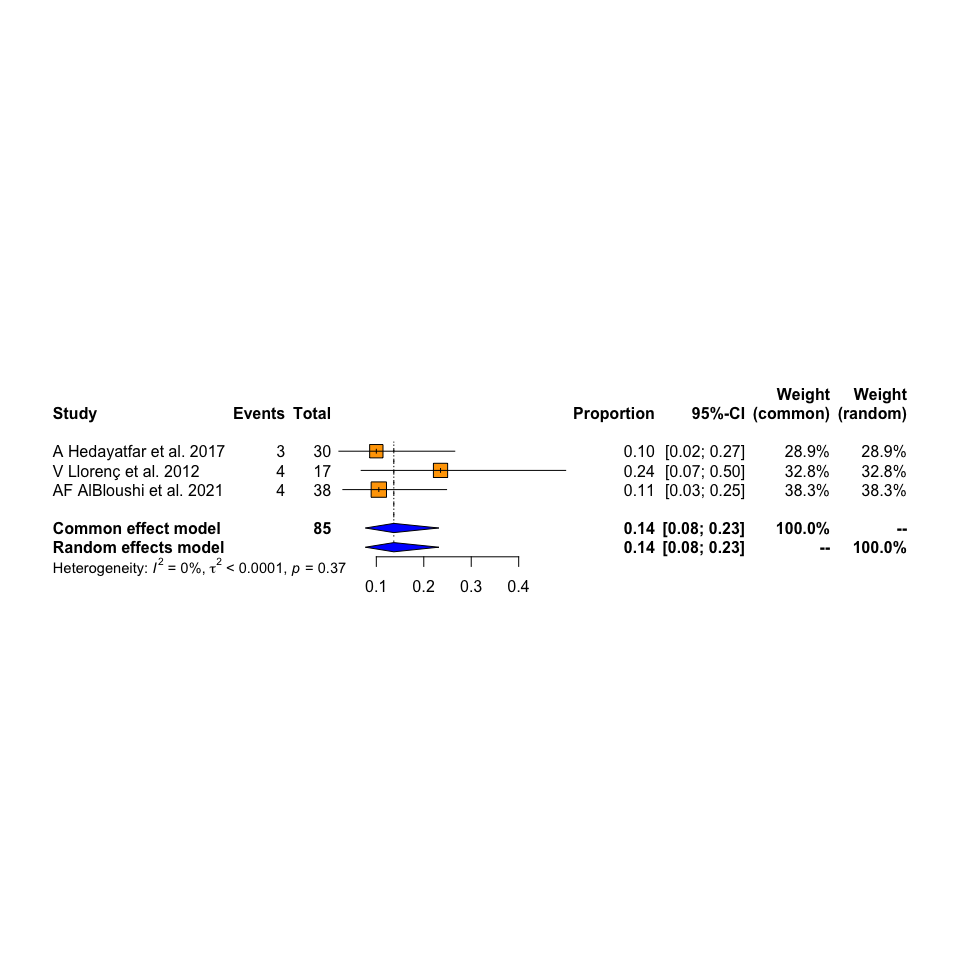


**S4 Fig 37: Prevalence of patients with posterior synechiae**


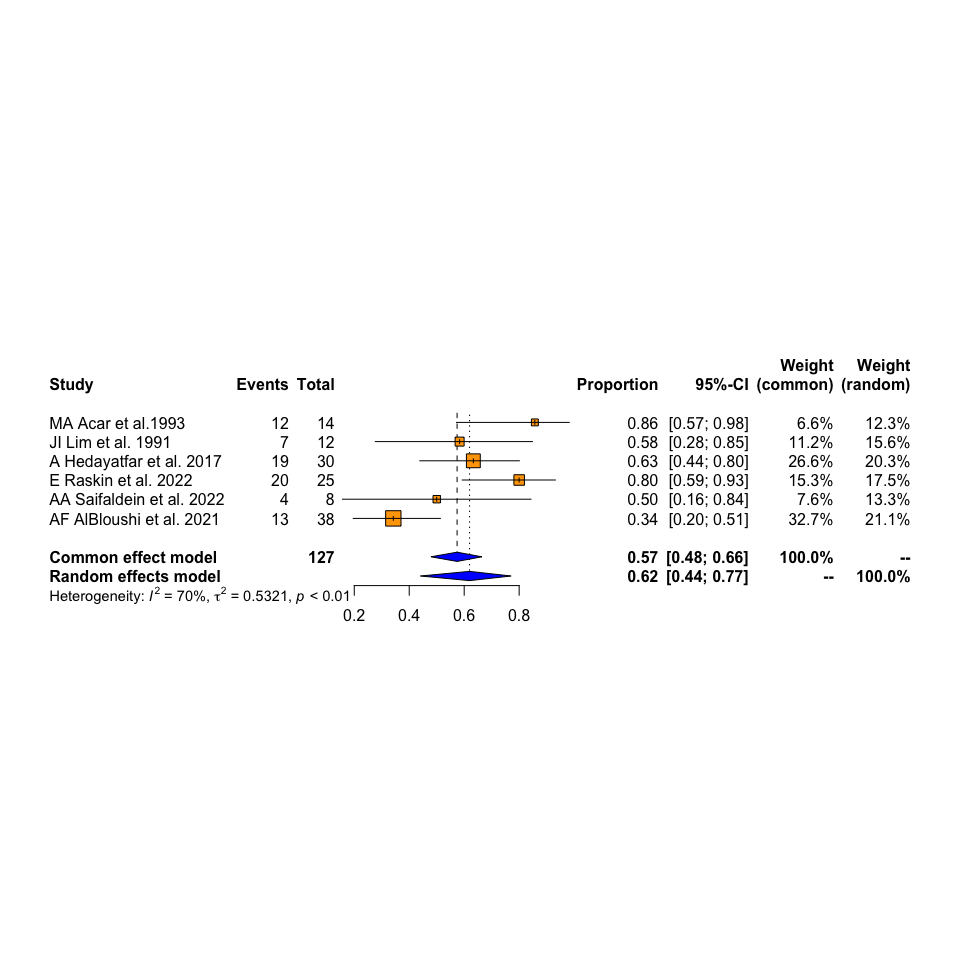


**Neurological clinical characteristics**

**S4 Fig 38: Prevalence of patients who presented neurological signs before uveitis**


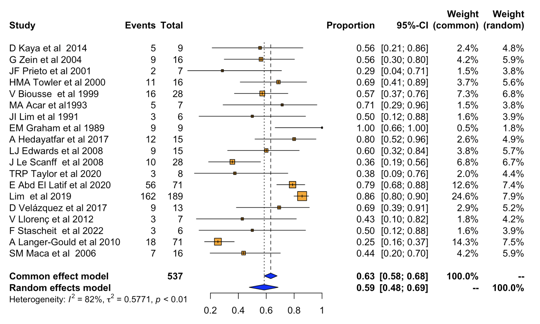


**S4 Fig 39 Prevalence of patients who presented neurological signs between 0-10 years.**


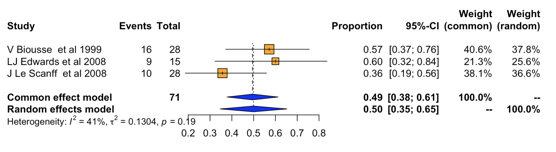


**S4 Fig 40: Prevalence of patients who presented neurological signs between 11-20 years.**


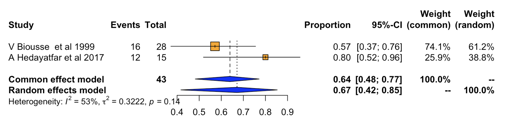


**S4 Fig 41: Prevalence of patients who presented neurological signs between 21-30 years.**


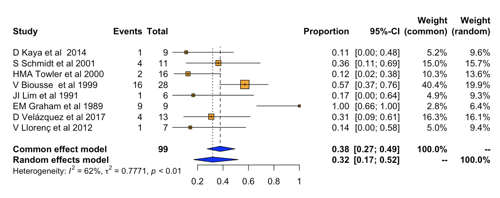


**S4 Fig 42: Prevalence of patients who presented neurological signs between 31-40 years.**


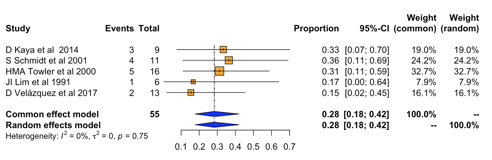


**S4 Fig 43: Prevalence of patients who presented neurological signs between 41-50 years.**


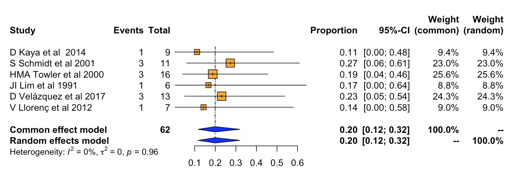


**S4 Fig 44: Prevalence of patients who presented neurological signs > 60 years.**


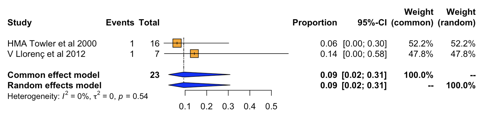


**S4 Fig 45: Prevalence of patients with relapsing-remitting multiple sclerosis**


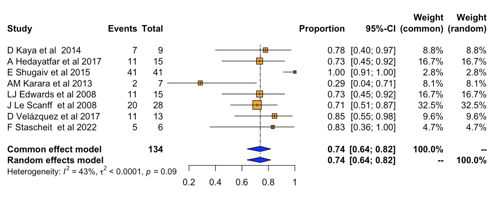


**S4 Fig 46: Prevalence of patients with primary progressive multiple sclerosis**


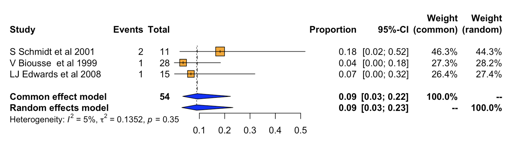


**S4 Fig 47: Prevalence of patients with secondary progressive multiple sclerosis**


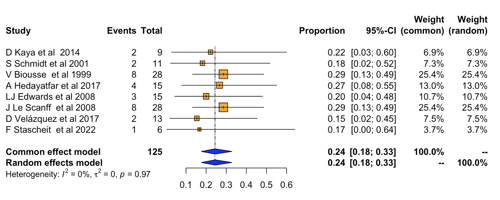


**S4 Fig 48: Prevalence of patients with supratentorial lesions in magnetic resonance imaging**


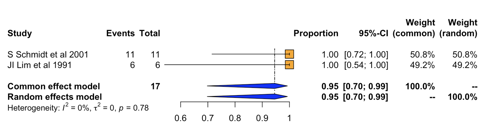


**S4 Fig 49: Prevalence of patients with spinal cord lesions in magnetic resonance imaging**


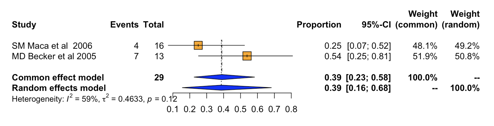


**Treatment**

**S4 Fig 50: Prevalence of patients treated with Beta Interferon**


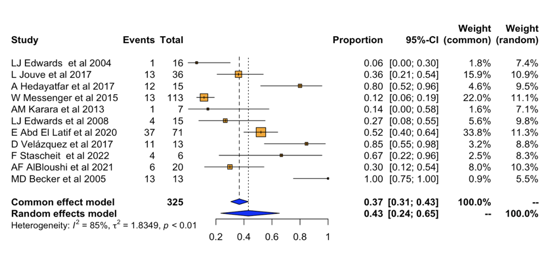


**S4 Fig 51: Prevalence of patients treated with Glatiramer.**


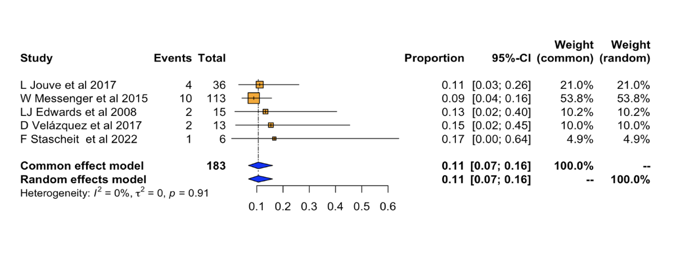


**S4 Fig 52: Prevalence of patients treated with Natalizumab.**


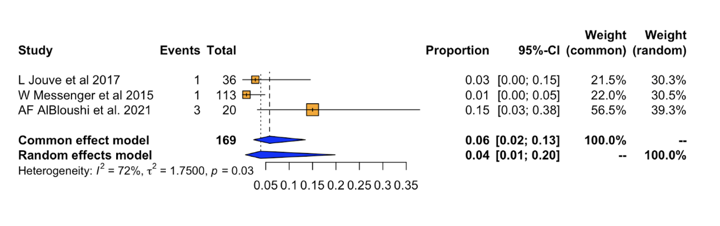


**S4 Fig 53: Prevalence of patients treated with Fingolimod.**


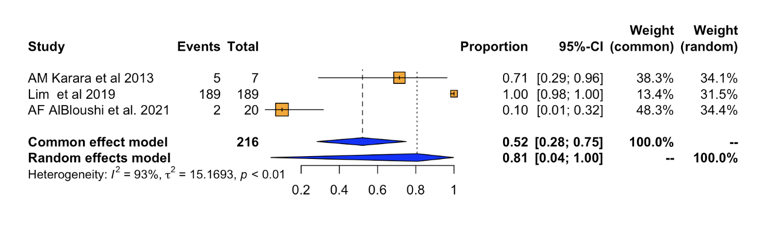


**S4 Fig 54: Prevalence of patients treated with Methotrexate.**


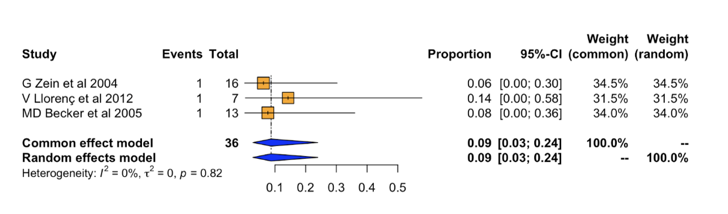


**S4 Fig 55: Prevalence of patients treated with Azathioprine**


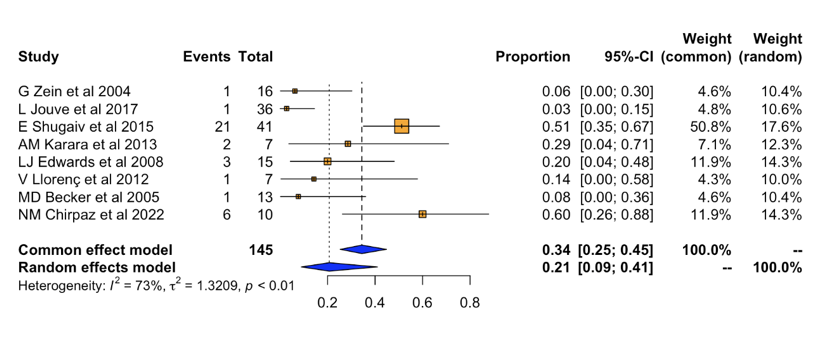


**S4 Fig 56: Prevalence of patients treated with Mycophenolate.**


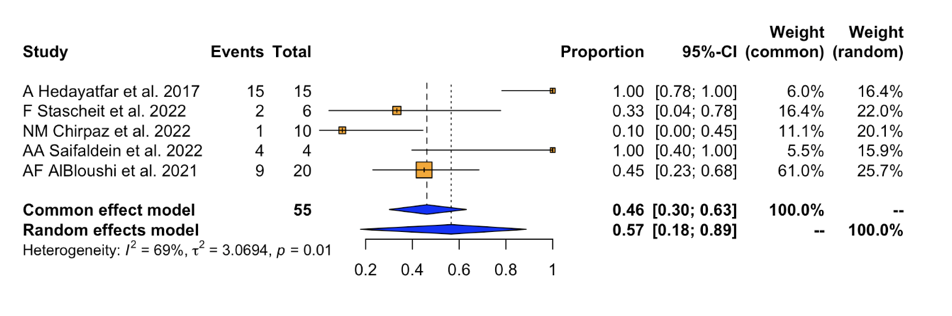


**S4 Fig 57: Prevalence of patients treated with Ciclosporine.**


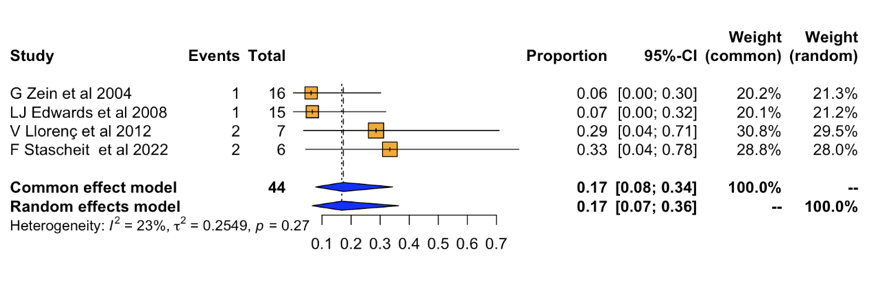


**S4 Fig 58: Prevalence of patients treated with Tacrolimus.**


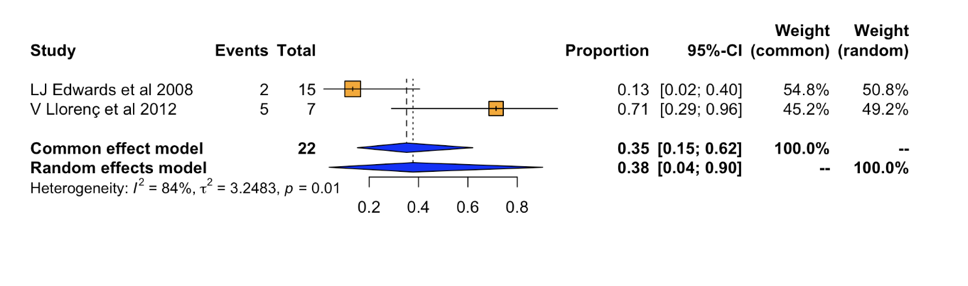


**S4 Fig 59: Prevalence of patients that required surgical intervention.**


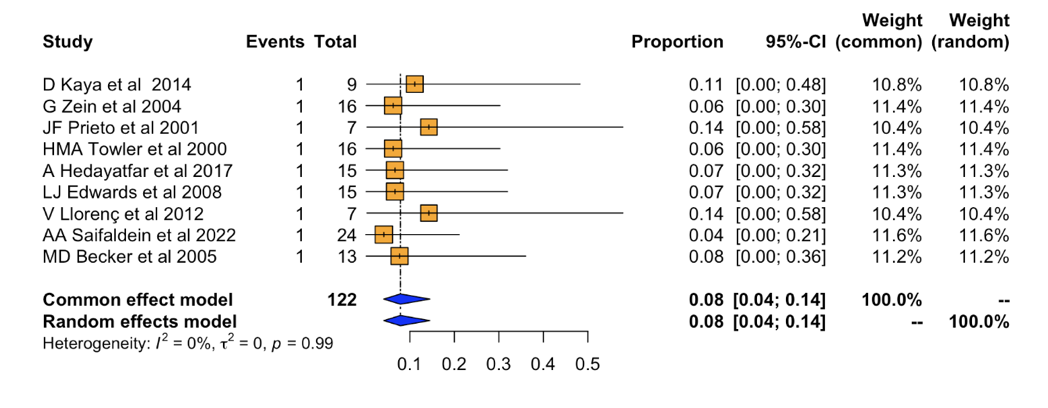


**Subgroup Metanalysis**

**S4 Fig 60: Male prevalence by continent**


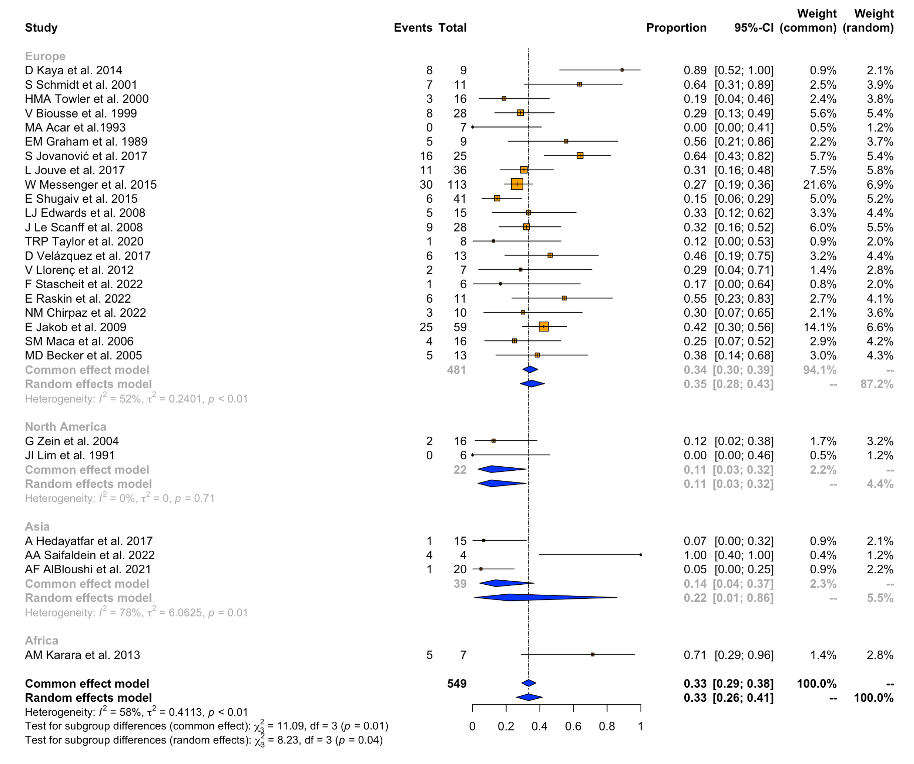

Supplement: S3 File — (DOCX) [file pone.0307455.s004.docx]
